# Markov Decision Process–Based Personalized Follow-Up Planning for Type 2 Diabetes: Retrospective Cohort Study

**DOI:** 10.2196/88206

**Published:** 2026-09-02

**Authors:** Silei Chen, Tianyi Liu, Zhonghua Sun, Jian Jia, Dong Hang, Wenhong Zhang

**Affiliations:** 1 School of Medicine Nanjing University Nanjing, Jiangsu China; 2 School of Business Nanjing University Nanjing, Jiangsu China; 3 Jiangsu Province Hospital Nanjing, Jiangsu China; 4 Department of Epidemiology, Jiangsu Key Lab of Cancer Biomarkers, Prevention and Treatment, Collaborative Innovation Center for Cancer Personalized Medicine School of Public Health Nanjing Medical University Nanjing, Jiangsu China

**Keywords:** type 2 diabetes, Markov decision process, personalized follow-up, cost-effectiveness, primary care, decision support

## Abstract

**Background:**

Personalized follow-up for type 2 diabetes may improve the alignment between monitoring intensity and patient needs, but operational approaches that jointly consider follow-up timing, modality, expected health benefits, and resource use remain limited.

**Objective:**

This study develops a Markov decision process (MDP) framework for personalized follow-up planning, externally validates complementary risk-prediction models, and estimates the projected 12-month cost-effectiveness of model-generated follow-up strategies.

**Methods:**

We retrospectively analyzed longitudinal data from 41,398 patients with type 2 diabetes managed in 10 community health centers in Nanjing, China, over the 2015-2024 calendar period. An independent Shanghai cohort included 25,506 patients from 10 communities. We developed least absolute shrinkage and selection operator (LASSO)-Cox models to predict 1-year incident complication and mortality risk and evaluated discrimination in Shanghai. Separately, a 12-cycle finite-horizon MDP used observed action-conditional state transitions with prespecified utility, cost, access, and willingness-to-pay parameters to generate personalized follow-up policies.

**Results:**

For an example patient initially without recorded complications (S0), the personalized policy increased annual effectiveness by 0.48 quality-adjusted life days (QALDs; 0.0013 quality-adjusted life years [QALYs]) and cost by 33.46 CNY (1 CNY=US $0.15), yielding an incremental cost-effectiveness ratio (ICER) of 25,499 CNY/QALY. In a heterogeneous simulated cohort of 1000 patients initialized in S0, mean annual effectiveness increased from 313.52 to 314.05 QALDs (0.85896 to 0.86041 QALYs), and mean annual cost increased by 24.67 CNY, yielding an ICER of 17,016 CNY/QALY. External C-indices were 0.778 for incident complications and 0.812 for mortality. Deterministic sensitivity analyses did not materially alter the cost-effectiveness conclusion.

**Conclusions:**

The MDP framework generated individualized 12-month follow-up policies with small projected QALY gains at modest incremental program cost, while the complementary risk models demonstrated external discrimination in an independent cohort. As action-conditional transitions were estimated from observational records and several economic parameters were prespecified, the modeled differences represent projections rather than causal treatment effects and require prospective implementation and economic validation before clinical adoption.

## Introduction

Diabetes is one of the most common chronic diseases globally [1]. In China, a combination of population growth [2], aging [3], rising obesity rates [4], and an increasingly sedentary lifestyle [5] has led to a substantial increase in the number of people with diabetes.

Diabetes management guidelines emphasize follow-up care as a critical nonpharmacological component of long-term disease management. This approach is important for monitoring glycemic control, identifying complications, improving quality of life, and supporting sustained self-management [6,7]. For instance, the American Diabetes Association recommends more frequent follow-up visits for newly diagnosed or poorly controlled patients, with individualized schedules for those at high risk of complications [8]. European guidelines also emphasize patient-centered care and individualized management according to patient characteristics, risk profiles, preferences, and treatment burden [9-11]. The Chinese guidelines recommend that primary health care providers conduct 4 evenly spaced face-to-face follow-up visits each year for patients diagnosed with type 2 diabetes [12].

Beyond guideline-scheduled visits, prior studies have examined follow-up frequency and regularity, telemedicine-assisted follow-up, and risk-stratified encounter schedules for diabetes and other chronic diseases [13-18]. These studies indicate that follow-up involves not only whether contact occurs, but also how often, when, and through which modality. However, only a few approaches provide an operational decision model that jointly determines follow-up timing and modality while accounting for projected health benefit, access burden, and resource use in primary care.

Implementing personalized follow-up in primary care is challenging because relevant longitudinal data are fragmented, planning often depends on manual judgment, and growing chronic-disease caseloads constrain clinical capacity. These constraints create a need for decision support that can translate routinely collected data into explicit follow-up policies while preserving clinical interpretability and resource awareness.

This study aimed to develop a Markov decision process (MDP)–based personalized follow-up planning framework for patients with type 2 diabetes, externally evaluate the discrimination of complementary risk-prediction models, and estimate the projected 12-month cost-effectiveness of model-generated personalized follow-up strategies compared with standardized follow-up.

## Methods

### Study Design

**Figure 1:**
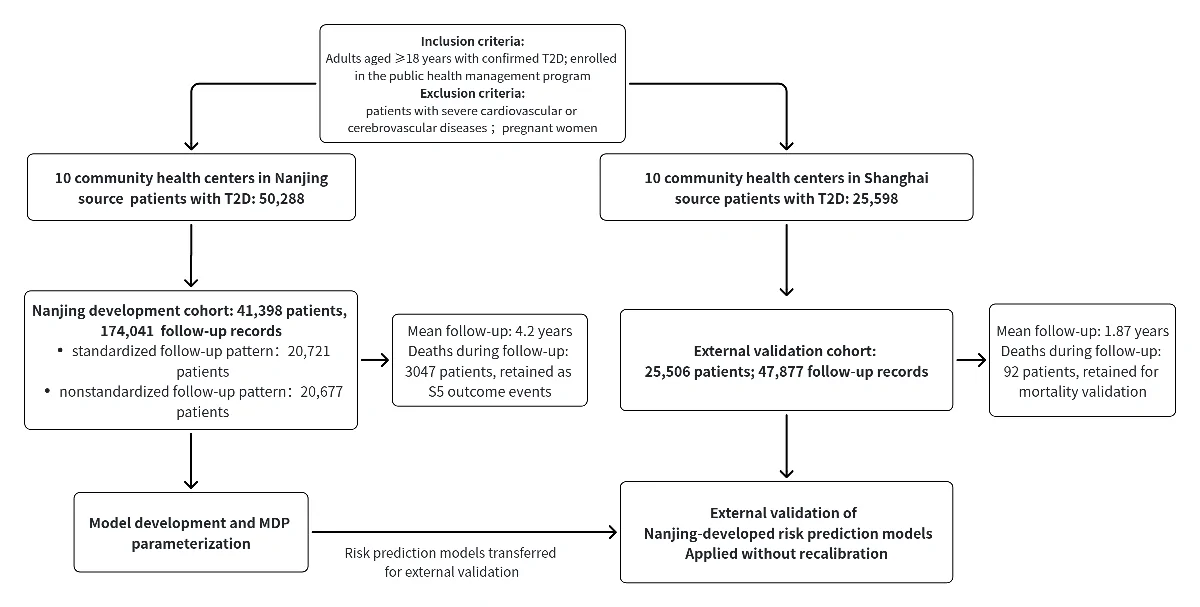
Flowchart of the Nanjing development cohort and Shanghai external validation cohort for Markov decision process (MDP)–based personalized follow-up planning in type 2 diabetes (T2D).

This study retrospectively analyzed real-world longitudinal data from 10 community health centers in Nanjing, China. The Nanjing source database covered the period 2015-2024, whereas individual patients had variable follow-up durations (Figure 1). Eligible patients were adults aged over 18 years with confirmed type 2 diabetes according to International Classification of Diseases, 10th Revision (ICD-10) codes and enrollment in the public health management program. Patients with severe cardiovascular or cerebrovascular diseases and pregnant women were excluded. A total of 50,288 individuals with type 2 diabetes were identified in the source database. After excluding individuals without analyzable longitudinal follow-up because of relocation, transfer out of the local public health management system, or incomplete or invalid records, 41,398 patients were included in the analytic cohort. Deaths observed after cohort entry were retained as outcome events and modeled as the absorbing state S5 rather than treated as loss to follow-up. The analytic cohort had a mean follow-up duration of 4.2 years and comprised 224 source data fields and 174,041 longitudinal follow-up records after analytic restructuring. Before risk-model development, identifiers, dates, per-measurement metadata, administrative and follow-up-process variables, outcome and survival-time variables, and fields used solely to construct MDP states and transitions were excluded from candidate prediction features. Eligible variables were subsequently encoded into 124 candidate design predictors as described below.

Patients were categorized into standardized and nonstandardized follow-up groups based on their observed follow-up patterns. Standardized follow-up was defined as 4 annual in-person visits, 1 per quarter, as stipulated by Chinese public health regulations. Nonstandardized follow-up was defined as any observed pattern that deviated from this guideline-based schedule, including fewer or more than 4 annual follow-ups, uneven follow-up intervals, or the use of telephone or community-based follow-up modalities instead of the scheduled in-person visits. The standardized follow-up group included 20,721 patients and the nonstandardized follow-up group included 20,677 patients. Because of the large sample size, several baseline variables showed statistically significant differences between the 2 groups; therefore, standardized mean differences were used as the primary criterion for assessing baseline comparability. Most measured baseline characteristics showed small between-group differences. No additional propensity score matching or weighting was performed because MDP transition-probability estimation required preserving the full observed state-action transition structure and avoiding sparse transition cells. The nonstandardized follow-up group was not a randomized intervention arm but an observational category derived from routine follow-up records. The comparison between standardized and nonstandardized follow-up patterns was treated as a descriptive assessment of observed follow-up patterns rather than as a causal estimate of treatment effect (Table 1).

**Table 1:** Baseline characteristics of patients with type 2 diabetes in the Nanjing development cohort by observed follow-up pattern.

Features			Standard follow-up group		Nonstandard follow-up group		*P* value		SMD^a,b^
Number of patients, n			20,721		20,677		N/A^c^		N/A
Age (years), mean (SD)			64.64 (10.63)		63.87 (11.05)		<.001		0.071
**Sex, n (%)**							.90		0.001
	Male	9948 (48.01)		9940 (48.07)					
	Female	10,773 (51.99)		10,737 (51.93)					
Duration of type 2 diabetes (years), mean (SD)			8.91 (6.86)		8.25 (6.98)		<.001		0.095
**Family history of type 2 diabetes, n (%)**							<.001		0.052
	Yes	6216 (30.00)		5716 (27.64)					
	No	14,505 (70.00)		14,961 (72.36)					
**Complications, n (%)**							.15		0.015
	Yes	354 (1.71)		315 (1.52)					
	No	20,367 (98.29)		20,362 (98.48)					
**Disability, n (%)**							.55		0.006
	Yes	940 (4.54)		912 (4.41)					
	No	19,781 (95.46)		19,765 (95.59)					
**Education level, n (%)**							<.001		0.027
	Illiterate or semiliterate	2528 (12.20)		2345 (11.34)					
	High school education or less	16,633 (80.27)		16,749 (81.00)					
	College education or greater	1560 (7.53)		1583 (7.66)					
**Types of medical insurance, n (%)**							.04		0.031
	Urban-employee medical insurance	11,872 (57.29)		11,737 (56.76)					
	Urban-resident medical insurance	8288 (40.00)		8334 (40.31)					
	Rural medical insurance	223 (1.08)		195 (0.94)					
	Other types of medical insurance	338 (1.63)		411 (1.99)					
**Smoking, n (%)**							<.001		0.039
	Yes	5867 (28.31)		5497 (26.59)					
	No	14,854 (71.69)		15,180 (73.41)					
**Drinking, n (%)**							.57		0.006
	Yes	4149 (20.02)		4093 (19.79)					
	No	16,572 (79.98)		16,584 (80.21)					
**Exercise frequency, n (%)**							<.001		0.199
	Almost never exercise	3572 (17.24)		4593 (22.21)					
	1-3 times per week	5317 (25.66)		6299 (30.46)					
	More than 3 times per week	11,832 (57.10)		9785 (47.32)					
**Tooth loss, n (%)**							.002		0.031
	Yes	9804 (47.31)		10,102 (48.86)					
	No	10,917 (52.69)		10,575 (51.14)					
**Lower-limb edema, n (%)**							.02		0.022
	Yes	5325 (25.70)		5517 (26.68)					
	No	15,396 (74.30)		15,160 (73.32)					
**Blood type, n (%)**							<.001		0.199
	A	5886 (28.41)		5484 (26.52)					
	B	5066 (24.45)		4461 (21.57)					
	O	5606 (27.05)		4841 (23.41)					
	AB	4163 (20.09)		5891 (28.49)					
**Blood pressure (mm Hg), mean (SD)**									
	Diastolic	80.06 (8.87)		80.01 (8.92)		.60		0.005	
	Systolic	134.58 (15.15)		135.12 (15.34)		<.001		0.036	
Waist measurement (cm), mean (SD)			85.45 (9.08)		85.81 (8.95)		<.001		0.040
BMI (kg/m^2^), mean (SD)			25.01 (3.16)		25.02 (3.11)		.87		0.002
Heart rate (times/min), mean (SD)			74.33 (9.22)		74.88 (9.47)		<.001		0.058
Urine microalbumin (mg/24 h), mean (SD)			41.96 (47.11)		39.70 (46.07)		<.001		0.048
Fasting blood glucose (mmol/L), mean (SD)			7.35 (2.12)		7.53 (2.31)		<.001		0.082
Hemoglobin (g/L), mean (SD)			139.64 (12.90)		139.77 (13.01)		.29		0.010
Leukocyte count (×10^9^/L), mean (SD)			6.33 (1.46)		6.30 (1.42)		.03		0.021
Platelet count (×10^9^/L), mean (SD)			202.81 (49.64)		203.75 (49.20)		.05		0.019
Total cholesterol (mmol/L), mean (SD)			4.88 (1.00)		4.89 (0.98)		.59		0.005
Triglycerides (mmol/L), mean (SD)			1.38 (0.57)		1.39 (0.57)		.02		0.023
Low-density lipoprotein cholesterol (mmol/L), mean (SD)			2.66 (0.80)		2.67 (0.80)		.08		0.017
High-density lipoprotein cholesterol (mmol/L), mean (SD)			1.40 (0.34)		1.41 (0.32)		.03		0.021

^a^SMD: standardized mean difference.

^b^SMD was used to assess between-group differences in baseline characteristics, with an SMD<0.10 indicating small differences between groups. Multicategory SMDs were calculated using the multivariate formula of Yang and Dalton [19] and refer to the categories as displayed in this table. Most SMDs were below 0.10. No additional propensity score matching or weighting was performed, because estimation of the Markov decision process state-action transition probabilities required preserving the full observed transition structure and avoiding sparse transition cells.

^c^N/A: not applicable.

External evaluation of the complementary risk-prediction models used an independent cohort of 25,506 patients from 10 communities in Shanghai, with a mean follow-up duration of 1.87 years (data collected in 2023-2024). Variables required for the primary risk models were harmonized in definition and coding across the Nanjing and Shanghai cohorts. The Shanghai cohort was used only to evaluate the external discrimination of the Nanjing-developed risk models and was not used to validate the MDP policy or cost-effectiveness projections.

### Ethical Considerations

This study was approved by the Ethics Board of Nanjing University, Nanjing, China (approval number OAP20240930001), and the approval covered the retrospective analysis of both the Nanjing development cohort and the Shanghai external validation cohort. The requirement for informed consent was waived because all data were deidentified and collected during routine clinical practice and public health management. No compensation was provided to participants, and no identifiable individual information is presented in this paper or its multimedia appendix.

### Model Development

We formulated personalized follow-up planning as a finite-horizon MDP with 12 monthly decision cycles. The model consisted of health states, follow-up actions, action-specific transition probabilities, and an immediate monthly net health benefit reward [20,21]. Table 2 summarizes the notation, state definitions, decision variables, health utility parameters, cost parameters, willingness-to-pay thresholds, and travel-related disutility parameters used in the model.

**Table 2:** Notation used in the 12-month Markov decision process model for personalized follow-up planning in type 2 diabetes.

Variables			Meaning statement
**Fundamental variables**			
	*i*	Patient, *i*=1, ..., *N*	
	*t*	Period, *t*=1, ..., 12	
	*s*_*i,t*_	Health state of patient *i* in month *t*, *s*_*i,t*_∈{S0, S1, S2, S3, S4, S5}	
	S0	No recorded diabetes-related complication	
	S1	Diabetic nephropathy	
	S2	Diabetic retinopathy	
	S3	Diabetic peripheral neuropathy	
	S4	Lower-extremity arterial disease and diabetic foot	
	S5	Death	
	*d*_*i,t*_	Patient *i* without complications at *t*, with 1 indicating yes and 0 indicating no.	
	*c*_any,__*i,t*_	Patient *i* has any recorded diabetes-related complication at *t*, with 1 indicating yes and 0 indicating no.	
	*C*_neph,__*i,t*_	Patient *i* has diabetic nephropathy at *t*, with 1 indicating yes and 0 indicating no.	
	*c*_ret,__*i,t*_	Patient *i* has diabetic retinopathy at *t*, with 1 indicating yes and 0 indicating no.	
	*c*_neuro,__*i,t*_	Patient *i* has diabetic peripheral neuropathy at *t*, with 1 indicating yes and 0 indicating no.	
	*c*_lead,__*i,t*_	Patient *i* has lower-extremity arterial disease and diabetic foot at *t*, with 1 indicating yes and 0 indicating no.	
	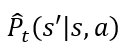	Action-specific transition probability of moving from health state *s* in month *t* to health state *s′* in month *t*+1 under follow-up action *a*.	
	Δ*t*	Length of each monthly cycle, fixed at 365/12 days in the base-case analysis.	
	*u*_*s*_	Health utility value of state *s*, where *s*=0, 1, 2, 3, 4, 5 corresponds to S0, S1, S2, S3, S4, and S5, respectively.	
	*c*_comp,__*s*_	Annual complication-related cost for health state *s*, where *s*=0, 1, 2, 3, 4, 5; the value is 0 for both S0 and S5.	
	*c*_base,__*i*_	Annual baseline diabetes management cost for patient *i*, applied only while the patient is not in the death state and converted to monthly cost by dividing by 12.	
	*c* ^0^_*t*_	Cumulative number of outpatient follow-up visits used before month *t*.	
	*F*_*i,t*_(*a*_*t*_, *c*^0^_*t*_)	Monthly modeled modality-specific follow-up cost for patient *i* in month *t*.	
	*f*_1_	Additional outpatient follow-up charge applied from the fifth outpatient visit onward.	
	*f*_2_	Modeled telephone follow-up charge per call.	
	*f*_3_	Modeled community follow-up charge per visit.	
	*c* ^base^_*i,t*_	Monthly baseline diabetes management cost for patient *i* in month *t*.	
	*c* ^comp^_*i,t*_	Monthly complication-related cost for patient *i* in month *t*.	
	*c* ^follow^_*i,t*_	Monthly modeled follow-up charge for patient *i* in month *t*.	
	*c* ^total^_*i,t*_	Monthly cost for patient *i* in month *t*, including monthly baseline cost, monthly complication-related cost, and the modeled follow-up-charge component.	
	*c* ^π^_*i*_	Annual cost for patient *i* under policy π, obtained by summing the 12 monthly total costs.	
	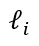	Travel distance of patient *i* to outpatient follow-up, measured in kilometers.	
	*Η*	Travel-related disutility coefficient applied to outpatient follow-up, defined as 0.00032 utility loss per kilometer in the base-case model.	
	*λ*_*D*_	WTP^a^ threshold expressed as CNY^b^/QALD^c^.	
	*λ*_*Y*_	WTP threshold expressed as CNY/QALY^d^.	
	*V*_*t*_(*s*,*c*^*O*^_*t*_; *λ*_*D*_)	Maximum expected cumulative net health benefit from month *t* onward.	
**Decision variables**			
	*a_t_*	Follow-up action selected in month *t*, where 0 denotes no follow-up, 1 outpatient follow-up, 2 telephone follow-up, and 3 community follow-up; the action set is *A*={0, 1, 2, 3}.	

^a^WTP: willingness-to-pay.

^b^1 CNY=US $0.15.

^c^QALD: quality-adjusted life day.

^d^QALY: quality-adjusted life year.

**Figure 2:**
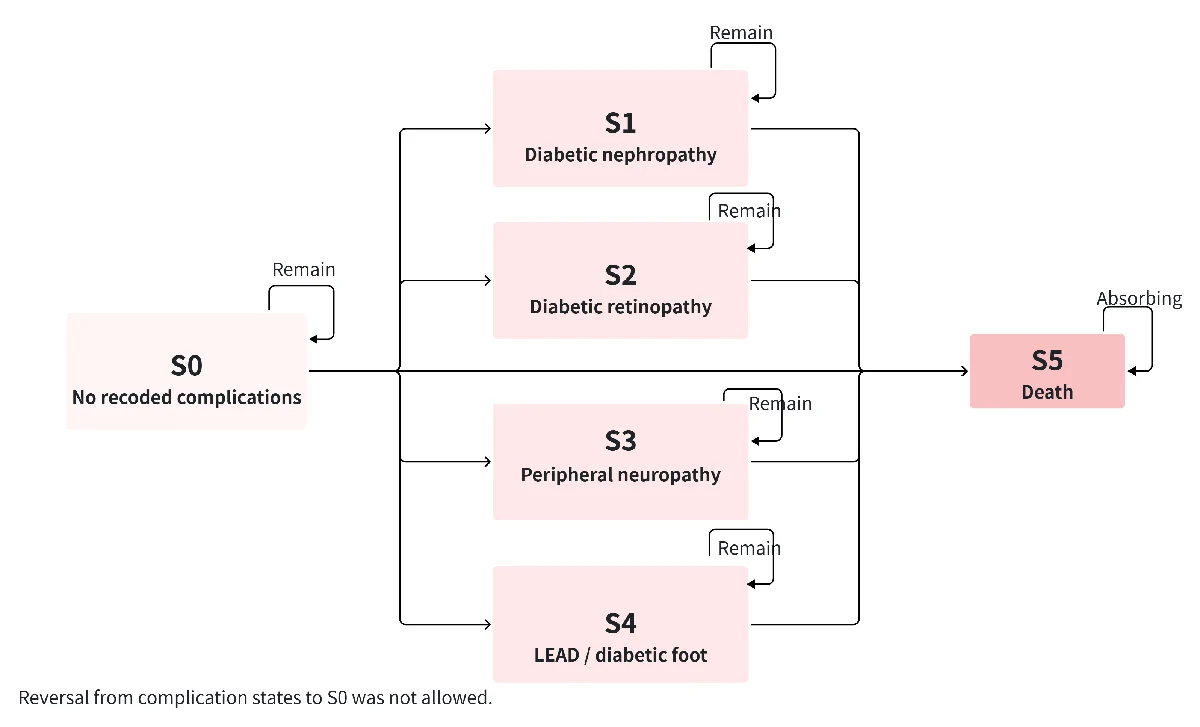
Health-state transition structure of the 12-month Markov decision process model for personalized follow-up planning in patients with type 2 diabetes. LEAD: lower-extremity arterial disease.

Health states were operationally defined using structured diagnosis records coded according to the ICD-10 Healthcare Security Version [22] issued by the National Healthcare Security Administration of China. Diabetes-related complication states were identified using ICD-10 code categories corresponding to type 2 diabetes with renal complications, ophthalmic complications, neurological complications, peripheral circulatory complications, and diabetic foot. The health state space consisted of 6 mutually exclusive states: no recorded diabetes-related complication (S0), diabetic nephropathy (S1), diabetic retinopathy (S2), diabetic peripheral neuropathy (S3), lower-extremity arterial disease and diabetic foot (S4), and death (S5; Figure 2). Baseline prevalent complications were defined as relevant ICD-10–coded diagnoses recorded before or at model entry, whereas incident complications were defined as the first newly recorded ICD-10–coded diagnosis of a complication category during follow-up among patients without that complication category at baseline. Death was identified from mortality records and modeled as the absorbing state. Once a patient entered a complication state, reversal to S0 was not allowed in the model. The detailed ICD-10 Healthcare Security Version coding algorithm and state-assignment rules are provided in Tables S19 and S21-S23 in Multimedia Appendix 1.

Actions were the decisions or measures that could be taken in each state and that influenced state transitions within the model. In the model constructed in this study, decisions involved determining the number of follow-ups, intervals, and methods for a patient over the course of a year. Periods were defined as *t*=1, 2, ..., 12, corresponding to the months of the year. Clinical events and follow-up records with irregular dates were assigned to the corresponding monthly cycle according to their recorded dates. Monthly state-action transitions were then constructed from the patient’s recorded health state and follow-up action within each monthly cycle.

Let *a_t_* denote the follow-up action selected in month *t*, where *a_t_*=0 represents no follow-up, *a_t_*=1 represents outpatient follow-up, *a_t_*=2 represents telephone follow-up, and *a_t_*=3 represents community follow-up. Let *c*^0^*_t_* denote the cumulative number of outpatient follow-up visits completed before month *t*.

Rewards were defined as immediate monthly net health benefits rather than as ratios of health benefits to costs. The MDP objective was to maximize the expected cumulative net health benefit over the 12-month horizon. Monthly health benefits were measured in quality-adjusted life days (QALDs), and monthly costs were converted into QALD-equivalent losses using the willingness-to-pay threshold.

For patient *i* in month *t*, current state *s*, action *a*, and prior follow-up count *c*^0^*_t_*, the adjusted health utility was defined as follows:

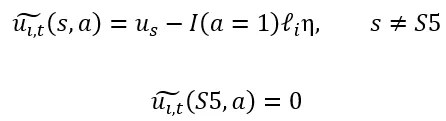



where *u_s_* is the health utility of state *s*, *I*(*a*=1) is an indicator for outpatient follow-up, 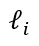 is the travel distance for outpatient follow-up, and η is the travel-related disutility coefficient. The death state S5 was assigned a utility of 0.

Monthly QALDs were calculated as follows:

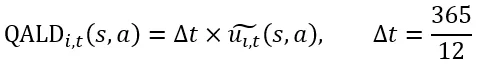



where Δ*t*=365/12 denotes the fixed length, in days, of each monthly cycle. Therefore, a patient who remains living throughout a complete cycle accrues the state-specific utility multiplied by 30.4167 days. Patients in the absorbing death state accrue 0 QALDs.

The costing analysis adopted a restricted public health program perspective rather than a full health-system or societal perspective. The annual baseline diabetes-management cost represented routine program expenditures, while modality-specific follow-up costs reflected additional charges in the model. The first 4 outpatient follow-up visits were assigned no additional charge because they are considered routine services under the public health program. This does not imply zero resource use or that all provider costs are fully captured by the baseline cost.

Monthly cost comprised a baseline management component, a complication-related component, and a follow-up component. The monthly follow-up cost for patient *i* in month *t*, denoted as *F_i,t_*(*a_t_*, *c^O^_t_*), was defined as follows:

*F_i,t_*(*a_t_*, *c^O^_t_*) = *I*(*a_t_*=1)*I*(*c^O^_t_*≥4)*f*_1_ + *I*(*a_t_*=2)*f*_2_ + *I*(*a_t_*=3)*f*_3_

where *I*(·) is an indicator function, *f*_1_ is the additional outpatient follow-up charge applied from the fifth outpatient visit onward, *f*_2_ is the modeled telephone follow-up charge per call, and *f*_3_ is the modeled community follow-up charge per visit. Under the specified accounting convention, the first 4 outpatient follow-up visits were assigned no additional modality-specific charge. This assignment represents a charging rule within the model and should not be interpreted as indicating zero provider resource use. Modality-specific charges were accrued only in the month in which the corresponding follow-up action occurred.

The monthly cost components, monthly total cost, and annual cost over the 12-month horizon were then calculated as follows:

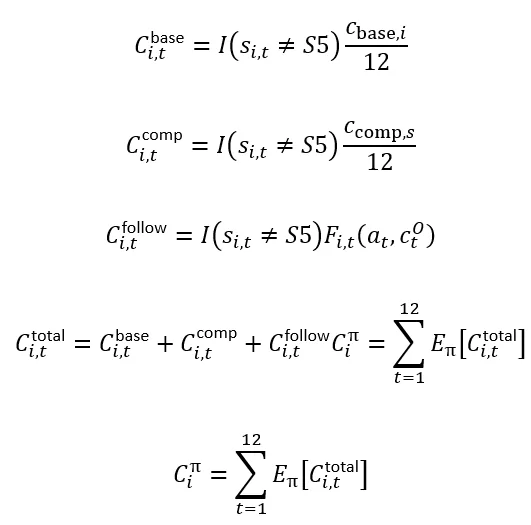



The annual baseline diabetes management cost for patient *i*, denoted by *c*_base,_*_i_*, was calculated using the following prespecified age-adjustment function:

*c*_base,_*_i_* = *c*_max_/[1 + exp(–Age*_i_*/*k*)]

where *c*_max_=15,000 CNY/year (1 CNY=US $0.15) was the annual baseline-management cost ceiling and *k*=10 was the age-scale parameter. Over the modeled age range of 40-80 years, this function generated annual baseline management costs of approximately 14,730-14,995 CNY. It therefore represented an approximately constant annual baseline diabetes-management cost with limited age adjustment and was treated as a prespecified modeling assumption rather than a cost function estimated directly from individual-level expenditure data. The baseline management cost was accrued in all living states S0-S4. Patients in states S1-S4 additionally accrued the corresponding annual complication-related cost *c*_comp,_*_s_*. As the MDP used monthly cycles, both annual cost components were divided by 12 before monthly accrual. No baseline management, complication-related, or follow-up cost was accrued after entry into S5. Thus, all annual costs reported in the cost-effectiveness analyses were obtained as the sum of the 12 monthly costs.

The willingness-to-pay threshold was expressed internally as CNY per QALD:

*λ_D_*=(*λ_Y_*/365)

where *λ_Y_* is the willingness-to-pay threshold expressed as CNY per quality-adjusted life year (QALY) and *λ_D_* is the corresponding threshold expressed as CNY per QALD.

The immediate reward used in the MDP was the monthly net health benefit:


ri,t(st,at,cOt; λD) = QALDi,t(st,at) – (Ctotali,t/λD)

Thus, the willingness-to-pay threshold entered the MDP optimization through the net health benefit reward function. Incremental cost-effectiveness ratios (ICERs) were calculated after policy optimization and were not maximized directly in the Bellman recursion. Transition probabilities were estimated from observed monthly transitions in the longitudinal data. To reduce instability arising from sparse state-action cells, we applied Laplace smoothing to the transition probability estimates. For month *t*, current state *s*, action *a*, and admissible next state *s*′, the smoothed transition probability was calculated as follows:

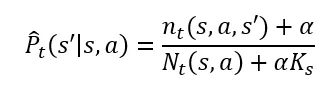



where *n_t_*(*s*, *a*, *s′*) is the observed number of transitions from *s* to *s′* under action *a* in month *t*, *N_t_*(*s*, *a*) is the total number of observed transitions starting from state *s* under action *a* in month *t*, and *K_s_* is the number of clinically admissible next states under the no-reversal constraint. The main analysis used *α*=1. Sensitivity analyses were conducted using alternative Laplace smoothing parameters. The transition structure comprised 288 month-by-action-by-state cells. Four cells had no observed transitions; all 4 were in the absorbing death state S5 in month 12, where only 1 next state was admissible, so the smoothed probability remained 1 and the absorbing structure was preserved. Among the 240 cells starting from a living state, the smallest observed denominator was 28 and the median was 1453; only 1 of these cells had a denominator below 30, and 24 had a denominator below 100. Telephone follow-up was the sparsest action, with a median denominator of 131.5. Across all 288 cells, including those starting from the absorbing death state, 17 cells had a denominator below 30 and 55 had a denominator below 100.

We further conducted deterministic 1-way sensitivity analyses to assess uncertainty in key cost-effectiveness parameters. Health utility values, complication-related medical costs, the annual baseline-management cost ceiling *c*_max_, outpatient follow-up cost, telephone follow-up cost, community follow-up cost, and travel-related disutility were varied by +20% or –20% from their base-case values. Specifically, *c*_max_ was evaluated at 12,000, 15,000, and 18,000 CNY/year; the base-case value remained 15,000 CNY/year. For these scenarios, the MDP was re-solved, and group-level QALYs, costs, ICERs, and incremental net monetary benefits were recalculated while holding all other parameters at their base-case values. As the willingness-to-pay threshold entered the MDP objective through the net health benefit reward function, analyses varying the willingness-to-pay threshold were interpreted as willingness-to-pay–dependent policy reoptimization scenarios rather than fixed-policy acceptance-threshold analyses. Specifically, the MDP was re-solved under willingness-to-pay thresholds corresponding to 1× and 3× China’s 2024 per capita gross domestic product per QALY. Therefore, changes in incremental QALYs, incremental costs, and ICERs across willingness-to-pay scenarios reflect changes in the optimized policy, not a recalculation of the ICER for a fixed policy.

As the follow-up planning problem had a fixed 12-month horizon, the optimal policy was solved using backward induction. Let *V_T_*_+1_(*s*, *C_T_*_+1_; λ*_D_*) denote the value function at month *t*, where the horizon is *t*=12 and *c^O^_t_* records the cumulative number of outpatient follow-up visits used before month *t* for cost accounting. The value function gives the maximum expected cumulative net health benefit from month *t* onward for a patient in state *s*, and the terminal value was set as follows:

*V_T_*_+1_(*s*, *C^O^_T_*_+1_; λ*_D_*) = 0

For each month, state, and follow-up count, the value function was recursively updated as follows:

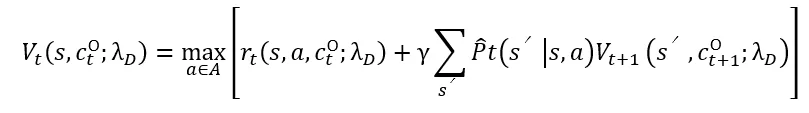



where *A*={0, 1, 2, 3} denotes the action set consisting of no follow-up, outpatient follow-up, telephone follow-up, and community follow-up; *r_t_*(*s, a,* *C^O^_t_*; λ*_D_*) is the immediate monthly net health benefit; *C^O^_t_*_+1_ is the updated follow-up count after action *a*; 
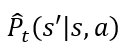
 is the smoothed transition probability; and *γ* is the discount factor. The base-case MDP used an algorithmic discount factor of *γ*=0.98 for the expected future value in the Bellman recursion, whereas annual QALDs and costs were reported as undiscounted sums of the 12 monthly outcomes. The discount factor was varied in deterministic sensitivity analyses, including *γ*=1.000 and *γ*=0.9959. At each monthly cycle, at most one action could be selected. Patients alive at the start of a monthly cycle accrued full monthly QALDs and costs, with transitions applied at the end of the cycle. The terminal value of zero closes the analysis at the 12-month horizon, so no health benefit accruing after month 12 is credited to any policy.

A monthly factor of 0.98 corresponds to an annual factor of 0.98^12^=0.785, that is, an effective annual discount rate of approximately 27%. This value was applied as an algorithmic parameter within the Bellman recursion and does not enter the reporting of effectiveness or costs: annual QALDs and costs are reported as undiscounted sums of the 12 monthly values. The monthly factor of 0.9959, corresponding to the annual rate of 5% recommended by the China Guidelines for Pharmacoeconomic Evaluations [23], and the undiscounted case of 1.000 were both examined in deterministic sensitivity analysis.

Clinical variables such as fasting blood glucose, blood pressure, BMI, urine microalbumin, complication indicators, and other electronic health record features were treated as candidate inputs for state identification and risk prediction. The least absolute shrinkage and selection operator (LASSO)-Cox models identified routinely collected variables that discriminate complication and mortality risk.

For the example-patient analysis, we first specified the patient’s baseline characteristics (Table 1) and assigned the patient to an initial health state according to baseline complication status. The MDP then generated a 12-month personalized follow-up policy for each possible initial state from S0 to S4. The primary analysis used exact expected-value propagation over the smoothed transition probabilities to calculate annual QALDs, annual costs, and ICERs. Under policy π, the annual expected QALDs for patient *i* were calculated by summing the expected monthly QALDs across all 12 cycles. Annual QALYs were obtained by dividing annual QALDs by 365.

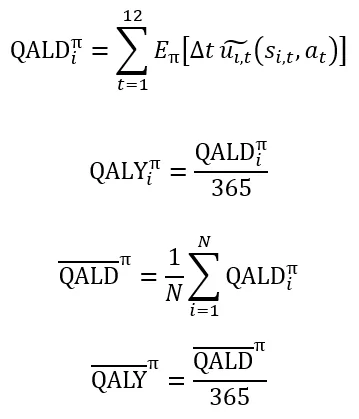



To check the simulation algorithm, we additionally performed a Monte Carlo simulation with 10,000 trajectories. The Monte Carlo analysis was used as an algorithmic check rather than as the primary estimator of QALDs and costs. The group-level simulation used a cohort of 1000 heterogeneous patients initialized in S0, with variation in age and travel distance. The simulated S0 cohort used a fixed random seed of 12; age was sampled as an integer from 40 to 80 years, and travel distance was sampled uniformly from 0.5 to 10.0 km. The state-specific base utility of S0 was fixed at 0.868 for all patients. Individual variation in adjusted utility arose from action-specific travel disutility and subsequent differences in expected health-state occupancy. A worked month-by-month expected-value trace for 1 selected simulated patient and direct reconciliation of monthly and annual QALDs and costs for selected patient-strategy examples are provided in Tables S8 and S9 in Multimedia Appendix 1.

For the economic evaluation, QALDs were retained as the model’s health-effect unit because the MDP used monthly cycles over a 12-month horizon. To align with standard health economic reporting, QALDs were converted to QALYs by dividing QALDs by 365, and ICERs were reported primarily as CNY per QALY in accordance with conventional cost-utility analysis and pharmacoeconomic evaluation practice [23-25]. CNY per QALD was provided only as a supplementary equivalent where applicable.

### Model Parameterization

#### Transition-Probability Parameterization

Empirical monthly state-action transition counts were constructed from observed longitudinal follow-up records. For the primary analysis, all eligible chronologically continuous month-to-month transitions were retained, including transitions crossing calendar-year boundaries, provided that the records represented consecutive monthly observations for the same patient. Clinical events and follow-up actions were assigned to their corresponding monthly cycles according to their recorded dates, and transitions were summarized by cycle month, current health state, follow-up action, and next health state. Laplace smoothing was then applied to these aggregated transition counts.

The aggregated transition table contained the observed transition count *nt*, *a*, *s*, *s′*, denominator *Nt*, *a*, *s*; number of clinically admissible next states *Kt, a, s*; admissible-transition indicator; and smoothed transition probability. The smoothed probabilities in this file were used to construct the transition tensor *P*[*t, a, s*, *s′*] for all primary analyses. The observation unit underlying these counts is the patient-month within an eligible patient-year, so that a patient contributing several eligible years contributes several monthly transitions; the denominators reported for each cell are therefore counts of patient-months rather than counts of distinct patients.

#### Missing Data and Imputation

We quantified missingness for selected core variables used in risk prediction, state identification, transition-probability estimation, and MDP parameterization; these summaries are reported in Table S18 in Multimedia Appendix 1. Records with missing outcome information, survival time, event indicators, or MDP-defining information such as health state or transition action were excluded from the corresponding analysis because these quantities define the analytic outcome or transition structure. Predictor imputation was performed only after the Nanjing development cohort and the independent Shanghai external validation cohort had been defined and kept separate. The preprocessing workflow is shown in Figure S1 in Multimedia Appendix 1.

For predictor variables retained in the modeling pipeline, missing values were handled using random-forest–based imputation. The imputation procedure for the Nanjing cohort was fitted within the development data. The Shanghai external validation cohort was processed separately without using outcome information or observations from the Nanjing model-fitting process, thereby reducing the risk of information leakage between development and external validation.

We considered the missing-data mechanism most consistent with missing at random, conditional on observed demographic characteristics, clinical measurements, visit patterns, and disease history. This assumption was considered more plausible than missing completely at random because missingness in real-world electronic health records is often related to clinical workflow and observed patient characteristics. However, missing not at random could not be fully excluded, particularly for laboratory tests or examinations that may have been selectively ordered for patients with higher clinical risk. As random-forest–based imputation does not fully propagate imputation uncertainty in the same way as multiple imputation, inferential statistics from imputed predictors were interpreted cautiously, and the prediction models were treated as risk-stratification tools rather than causal explanatory models.

#### Risk Prediction Model Development and Validation

The framework has 2 components with distinct roles. The risk prediction component, described in this subsection, evaluates whether routinely collected primary-care variables discriminate incident complications and mortality, and it is the component for which validation in an independent external cohort was possible. The decision component requires action-conditional transition probabilities, that is, the probability of each next health state given the current state and the follow-up action selected in that cycle. These cannot be obtained from the Cox models, which estimate 1-year risk from eligible person-year records and were not conditioned on the follow-up action; they were therefore estimated nonparametrically from the observed month-by-state-by-action transition counts, as described above. The 2 components are accordingly reported together but were not coupled numerically: no quantity produced by the risk prediction models enters the transition tensor, adjusted health utility, cost function, immediate reward, or Bellman recursion. Within the MDP, a policy is individualized by the current health state, travel distance, and age.

To predict the time from a patient’s current health state to subsequent complications or death, we developed 2 risk prediction models: an incident complication model and a mortality model. Both models were fitted on a person-year data structure: each patient contributed 1 record per eligible follow-up year, and the time variable within each record was the number of days of exposure during that year, censored at 365 days. The 2 models therefore estimate 1-year risk on the same timescale as the 12-month horizon of the MDP. For the incident complication model, the event indicator was a first newly recorded complication diagnosis within the year; patients whose first eligible year already carried a complication diagnosis were excluded as prevalent cases, and person-years occurring after a first complication were removed from the risk set, so that each patient contributed at most 1 incident event. For the mortality model, the event indicator was death within the year. As each patient contributed several person-years, all inference accounted for within-patient clustering: cross-validation folds were assigned at the patient level, the optimism bootstrap resampled patients rather than person-years and refitted the Cox model conditional on the LASSO-selected predictor set, and the refitted Cox models used cluster-robust SEs with the patient as the clustering unit. All candidate predictors were first entered into a LASSO-penalized Cox proportional hazards model for feature selection. The penalty parameter was tuned using 10-fold cross-validation based on partial-likelihood deviance. The lambda.1se rule was prespecified before model fitting as the primary tuning rule to obtain a parsimonious and stable prediction model, and the lambda.min rule was prespecified as a secondary analysis reported in Table S24 in Multimedia Appendix 1. Models were not selected on the basis of external validation performance. After predictors with nonzero LASSO coefficients were selected, these variables were refitted in multivariable Cox models to estimate coefficients, hazard ratios, and 95% CIs. As variable selection was performed before Cox refitting, *P* values from the refitted Cox models were interpreted as exploratory results only. The purpose of these models was prediction and risk stratification rather than causal inference. Model discrimination was assessed using the apparent C-index, the patient-level bootstrap optimism–corrected C-index conditional on the LASSO-selected predictor set, and the external C-index in the Shanghai validation cohort. The Cox proportional hazards assumption was assessed using scaled Schoenfeld residuals.

To assess whether departures from the proportional hazards assumption materially affected discrimination, particularly for the mortality model, we conducted an additional sensitivity analysis using random survival forest models, which are nonparametric ensemble methods for right-censored survival data and do not require the proportional hazards assumption [26]. For each outcome, the random survival forest model was trained using the same LASSO-selected predictors as the primary Cox model. Each forest used 500 trees, a minimum node size of 15, the log-rank splitting rule, and mtry=floor(sqrt[*p*]), where *p* was the number of LASSO-selected predictors. The same Nanjing development cohort and Shanghai external validation cohort were used, and discrimination was evaluated using the Harrell C-index. This sensitivity analysis examined whether relaxing the proportional hazards assumption materially changed model discrimination rather than replacing the LASSO-Cox models used for parsimonious risk stratification and interpretability.

The health utility values for patient *i* at time *t* are shown in Table 3. Published utility evidence for type 2 diabetes and diabetes-related complications indicates that the health utility values used in the model were 0.868 for S0 (no recorded diabetes-related complication), 0.753 for S1 (diabetic nephropathy), 0.830 for S2 (diabetic retinopathy), 0.769 for S3 (diabetic peripheral neuropathy), and 0.692 for S4 (lower-extremity arterial disease and diabetic foot); the utility value for S5 (death) was set to 0 [27,28].

**Table 3:** Health utility values used for type 2 diabetes and diabetes-related complication states in the Markov decision process model^a^.

Markov decision process state	Health state	Utility parameter	Health utility value
S0	No recorded diabetes-related complication	*u*_0_	0.868
S1	Diabetic nephropathy	*u*_1_	0.753
S2	Diabetic retinopathy	*u*_2_	0.830
S3	Diabetic peripheral neuropathy	*u*_3_	0.769
S4	Lower-extremity arterial disease and diabetic foot	*u*_4_	0.692
S5	Death	*u*_5_	0.000

^a^The state indices used in this table are identical to the Markov decision process health-state definitions and the analysis code: S1 denotes diabetic nephropathy, S2 diabetic retinopathy, S3 diabetic peripheral neuropathy, S4 lower-extremity arterial disease and diabetic foot, and S5 death.

Travel distance was included to represent the accessibility burden of outpatient follow-up. Prior evidence suggests that longer driving distance to primary care is associated with poorer glycemic control among patients with diabetes [29]. In the MDP, this burden was represented as a travel-related utility decrement applied only to outpatient follow-up. The base-case coefficient was 0.00032 utility loss per kilometer; it was treated as a model parameter rather than an exact causal estimate and was varied by +20% or –20% (range 0.000256-0.000384) in deterministic sensitivity analysis. No travel-related disutility was applied to nonoutpatient actions, and S5 (death) had a utility of 0.

Annual medical cost parameters are presented in Table 4. These annual costs were used as input parameters for the economic evaluation but were converted to monthly state costs before being applied in the 12-month MDP model.

**Table 4:** Annual medical cost parameters used for type 2 diabetes and diabetes-related complication states^a^.

MDP^b^ state	Health state	Cost parameter	Annual cost, CNY^c^/year
S0-S4 while alive	Baseline diabetes management	*c*_base__*,i*_	15,000/(1+exp[–Age_*i*_/10])
S0	No complication-related cost	*c*_comp__*,*__0_	0
S1	Diabetic nephropathy	*c*_comp,1_	12,000
S2	Diabetic retinopathy	*c*_comp,2_	4500
S3	Diabetic peripheral neuropathy	*c*_comp,3_	5500
S4	Lower-extremity arterial disease and diabetic foot	*c*_comp,4_	8000
S5	Death	N/A^d^	0

^a^*c*_base_*_,i_* denotes the annual baseline diabetes management cost accrued while patient *i* remained alive and was calculated as 15,000/(1+exp[–Age*_i_*/10]) CNY/year. The baseline management cost was accrued in all living states S0-S4 and was distinct from the state-specific annual complication-related cost *c*_comp_*_,s_*. Patients in S1-S4 therefore accrued both the baseline management cost and the corresponding complication-related cost. Annual costs were divided by 12 before entering the monthly MDP cycles. No cost was accrued after entry into S5.

^b^MDP: Markov decision process.

^c^1 CNY=US $0.15.

^d^N/A: not applicable.

To avoid ambiguity in state-to-parameter mapping, all utility and cost parameters were indexed according to the same state definitions used in the MDP health-state space. Specifically, S1 corresponded to diabetic nephropathy, S2 to diabetic retinopathy, S3 to diabetic peripheral neuropathy, S4 to lower-extremity arterial disease and diabetic foot, and S5 to death. The same mapping was used in the analysis code, parameter tables, sensitivity analyses, and ICD-10 state-assignment appendix. The complete state-to-parameter mapping is provided in Table S20 in Multimedia Appendix 1.

According to the Chinese National Basic Public Health Service Standards [30], patients with type 2 diabetes are entitled to 4 free outpatient clinic follow-up visits annually [12,31,32]. Under the model’s accounting convention, these first 4 outpatient visits were therefore assigned no additional modality-specific charge. Outpatient visits beyond the first 4 were assigned a charge of 12 CNY per visit, based on a registration fee of 2 CNY and a blood glucose test fee of 10 CNY. Telephone and community follow-up were assigned modeled charges of 13.8 CNY per call and 40 CNY per visit, respectively. These amounts should be interpreted as charge-based model inputs rather than estimates of the full economic opportunity cost of provider time, facilities, consumables, or administrative support. As the MDP used monthly decision cycles, these charges were accrued only in the month in which the corresponding follow-up action occurred.

## Results

### Risk Prediction Model Performance

For the incident-complication model, after excluding 669 patients with prevalent complications, removing 19,144 postevent person-years, and applying the prespecified outcome-time eligibility criteria, 152,415 eligible patient-year observations from 40,729 patients were included, with 7050 first incident complication events. After prespecified exclusions and one-hot encoding, 124 candidate design predictors entered the LASSO-Cox model; the lambda.1se rule retained only duration of diabetes. The apparent, optimism-corrected, and Shanghai external C-indices were 0.777, 0.777, and 0.778, respectively. The mortality model included 174,041 eligible patient-year observations from 41,398 patients and 3047 deaths. Of the same 124 candidate design predictors, 7 were retained by lambda.1se; the corresponding apparent, optimism-corrected, and external C-indices were 0.768, 0.767, and 0.812. Selected predictors are shown in Table 5.

**Table 5:** Penalized Cox model predictors for complication and mortality risk in the Nanjing development cohort^a^.

Risk factors		Coefficient	Hazard ratio (95% CI)	*P* value
**Outcome** **:** **complications**				
	Duration of diabetes	0.084	1.087 (1.084-1.090)	<.001
**Outcome** **:** **death**				
	Splenomegaly (abnormal)	0.468	1.597 (1.446-1.764)	<.001
	Lips (abnormal)	0.431	1.539 (1.393-1.699)	<.001
	Lower limb edema	0.395	1.484 (1.348-1.635)	<.001
	Mass	0.383	1.466 (1.325-1.622)	<.001
	Hepatomegaly	0.347	1.415 (1.271-1.575)	<.001
	Mobile dullness	0.274	1.315 (1.185-1.460)	<.001
	Age	0.070	1.072 (1.068-1.076)	<.001

^a^CIs and *P* values were obtained using cluster-robust SEs with the patient as the clustering unit. As predictor selection preceded Cox refitting, *P* values are exploratory and were not used for model selection.

In the prespecified lambda.min secondary analysis, 65 predictors were retained for incident complications and 95 for mortality. External C-indices were 0.752 and 0.833, respectively, compared with 0.778 and 0.812 for the primary lambda.1se models. Four selected categorical variables (occupation, marital status, medical payment type, and blood group) were structurally unavailable in Shanghai and, for the lambda.min external-discrimination analyses only, were fixed at their development-cohort reference levels. Predictor imputation was completed separately within the Nanjing development cohort and the Shanghai external-validation cohort before model fitting, and no additional imputation was performed during model fitting or external scoring. Neither primary lambda.1se model included these variables, so primary external validation was unaffected. In single-year cross-sectional analyses, duration of diabetes alone yielded C-indices of 0.790 in the second follow-up year (32,923 patients; 1194 events) and 0.768 in the third follow-up year (25,564 patients; 1383 events). These secondary analyses are reported in Table S24 in Multimedia Appendix 1.

Scaled Schoenfeld residual tests showed no evidence of violation of the proportional hazards assumption for the incident-complication model (global *P*=.93), whereas significant deviations were detected for the mortality model (global *P*<.001). Therefore, we used the LASSO-Cox models primarily as prediction and risk-stratification tools rather than as causal explanatory models. To examine whether relaxing the proportional hazards assumption materially affected model discrimination, we conducted a sensitivity analysis using random survival forest models that do not rely on the proportional hazards assumption. For the incident-complication model, the 500-tree random survival forest achieved a development C-index of 0.781 and an external C-index of 0.776, compared with 0.777 and 0.778 for the LASSO-Cox model, respectively. For the mortality model, the random survival forest achieved a development C-index of 0.791 and an external C-index of 0.814, compared with 0.768 and 0.812 for the LASSO-Cox model, respectively. The corresponding external C-index differences relative to LASSO-Cox were −0.002 for incident complications and +0.002 for mortality. These differences were negligible in magnitude, indicating that the random survival forest did not materially improve external discrimination. For mortality, the detected proportional-hazards deviations did not materially weaken discrimination; for incident complications, the random survival forest served as a nonparametric sensitivity check despite no detected proportional-hazards violation. We retained the LASSO-Cox models as the primary risk models because they provide a parsimonious and interpretable set of predictors. Detailed results of the random survival forest sensitivity analysis are provided in Table S17 in Multimedia Appendix 1.

### Development of Personalized Follow-Up Planning

The MDP model generated a 12-month personalized follow-up policy for each example patient according to the patient’s initial health state, age, and travel distance. Clinical variables from electronic health records were used for state identification and in the risk prediction models. The MDP itself used the current health state, age, monthly health utility, travel distance, and smoothed transition probability matrices to determine the optimal follow-up schedule and modality.

Table 6 summarizes the MDP-generated personalized follow-up strategies for example patients entering the model from different initial health states. The table reports the annual number of follow-ups and the distribution of follow-up modalities; the complete month-by-month schedules are provided in Table S1 in Multimedia Appendix 1. Patients in S5 were assigned no follow-up because death was modeled as an absorbing state. If a patient’s complication status changed during follow-up, the subsequent strategy would be updated according to the new health state. The illustrative S0 patient was aged 60 years and had a travel distance of 2.0 km. Group-level expected follow-up counts are reported separately in Table S11 in Multimedia Appendix 1.

**Table 6:** Summary of MDP^a^-generated personalized follow-up strategies by initial health states^b^.

Initial state	State definition	Total follow-ups per year, n	Outpatient, n	Telephone, n	Community, n	Follow-up pattern
S0	No complication	5	4	1	0	4 outpatient follow-ups and 1 telephone follow-up
S1	Diabetic nephropathy	8	8	0	0	8 outpatient follow-ups
S2	Diabetic retinopathy	6	6	0	0	6 outpatient follow-ups
S3	Diabetic peripheral neuropathy	6	6	0	0	6 outpatient follow-ups
S4	Lower-extremity arterial disease and diabetic foot	6	5	0	1	5 outpatient follow-ups and 1 community follow-up

^a^MDP: Markov decision process.

^b^The table summarizes the 12-month MDP-generated follow-up plans for example patients by initial health state. The complete month-by-month follow-up schedules are provided in Table S1 in Multimedia Appendix 1. Death was modeled as the absorbing state S5 and was therefore not assigned follow-up.

### Effectiveness at the Individual Level

At the individual level, the personalized follow-up strategy improved expected health outcomes for the example patient. For the example patient initially in S0, the personalized strategy increased annual QALDs from 313.64 to 314.12, corresponding to an increase of 0.48 QALDs per year. A Monte Carlo algorithmic check with 10,000 simulated trajectories produced similar results: the personalized strategy yielded 314.26 QALDs compared with 313.75 QALDs under standardized follow-up. In this check, the final complication-state probability was 5.19% under the personalized strategy and 5.09% under the standardized strategy, and the corresponding final death probabilities were 1.28% and 1.61%. These Monte Carlo estimates are reported in Table S4 in Multimedia Appendix 1. Complete expected-value effectiveness results across all initial states are provided in Table S2 in Multimedia Appendix 1.

### Cost-Effectiveness Analysis at the Individual Level

Annual costs were obtained by accumulating monthly state and follow-up costs over the 12-month horizon. For the example patient initially in S0, the personalized strategy increased effectiveness by 0.48 QALDs and increased annual cost by 33.46 CNY. The resulting ICER was 25,499 CNY/QALY, or 69.86 CNY/QALD. The Monte Carlo algorithmic check produced broadly consistent cost-effectiveness results for the S0 example patient. The willingness-to-pay threshold, set at 3 times China’s 2024 per-capita gross domestic product, was 287,248 CNY/QALY. The ICER was substantially below this threshold, indicating that the personalized follow-up strategy was cost-effective for the example patient under the model assumptions, although it was not cost-saving under the base-case program-costing assumptions. The complete individual-level cost-effectiveness results across initial health states are provided in Table S3 in Multimedia Appendix 1.

### Effectiveness at the Group Level

At the group level, we evaluated the MDP-based personalized follow-up strategy in a heterogeneous simulated cohort of 1000 patients initialized in S0. The analysis used a fixed cycle duration of 365/12 days for each of the 12 monthly cycles. The personalized strategy produced a mean annual effectiveness of 314.05 QALDs (0.86041 QALYs), compared with 313.52 QALDs (0.85896 QALYs) under the standardized strategy. The mean incremental effectiveness was 0.529 QALDs (0.00145 QALYs) per patient-year. The cohort-mean month-by-month accumulation of QALDs and costs under both strategies is reported in Table S10 in Multimedia Appendix 1; the 12 monthly values sum exactly to the annual cohort means reported in Tables S5 and S6 in Multimedia Appendix 1. The corresponding annual reconciliation based on patient-level and monthly sums is provided in Table S7 in Multimedia Appendix 1.

The cohort-level QALDs were consistent with the model’s state utilities and end-of-horizon state distributions. For reference, a patient remaining in S0 throughout the full year without outpatient travel disutility would accrue 365 × 0.868 = 316.82 QALDs. The slightly lower cohort means reflected transitions to lower-utility complication states, mortality, and outpatient travel disutility.

We also compared monthly expected state distributions under the 2 strategies, including complication-state occupancy and cumulative mortality, as reported in Tables S12 and S13 in Multimedia Appendix 1. By the end of the 12-month horizon, the expected proportion of patients occupying complication states S1-S4 was 5.114% under the personalized strategy and 5.115% under the standardized strategy, corresponding to 51.14 and 51.15 expected patients per 1000 patients, respectively. The corresponding expected cumulative mortality proportions were 1.437% and 1.610%, corresponding to 14.37 and 16.10 expected deaths, respectively. The 2 strategies therefore produced broadly comparable end-of-horizon state distributions, and the group-level advantage of the personalized strategy lay in improved QALDs and cost-effectiveness rather than in a large reduction in end-of-horizon complication or death probabilities. The personalized strategy nevertheless resulted in slightly lower cumulative mortality and 0.0126 additional expected living patient-months per patient over the 12-month horizon.

In the present group-level simulation, patients were initialized in S0 to evaluate personalized follow-up planning among patients without baseline complications. The base utility of S0 was fixed at 0.868 for all patients, whereas age, travel distance, and action-specific travel disutility contributed to heterogeneity in simulated outcomes. The personalized strategy generated a mean of 2.72 follow-ups per patient-year, including 1.74 outpatient follow-ups, 0.96 telephone follow-ups, and 0.01 community follow-ups; the standardized strategy generated 3.98 outpatient follow-ups per patient-year. The corresponding group-level strategy summary is provided in Table S11 in Multimedia Appendix 1.

### Cost-Effectiveness Analysis at the Group Level

Under the specified program-costing assumptions, the personalized strategy increased mean annual cost by 24.67 CNY and mean annual effectiveness by 0.00145 QALYs compared with standardized follow-up. The resulting ICER was 17,016 CNY/QALY, which was substantially below the willingness-to-pay threshold of 287,248 CNY/QALY. The complete group-level cost-effectiveness results are provided in Table S5 in Multimedia Appendix 1.

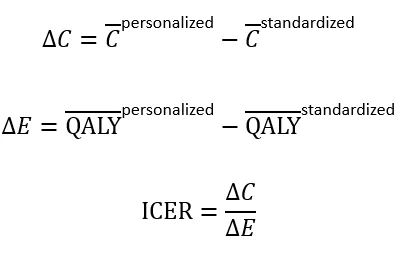



The incremental total cost was decomposed into baseline diabetes-management expenditure, complication-related cost, and additional modality-specific follow-up charges. Compared with standardized follow-up, the personalized strategy increased mean baseline diabetes-management expenditure by 15.64 CNY and mean additional follow-up charges by 14.94 CNY, while reducing mean complication-related cost by 5.91 CNY. These components reconciled exactly with the total incremental cost of 24.67 CNY. The higher baseline diabetes-management expenditure was consistent with the additional 0.0126 expected living patient-months under the personalized strategy. The additional follow-up charge reflected the expected distribution of outpatient, telephone, and community follow-up actions across patients and health-state trajectories.

### Sensitivity Analysis

To examine whether the MDP results were sensitive to the smoothing assumptions used in transition probability estimation, we repeated the group-level MDP analysis using alternative Laplace smoothing parameters of α=.1, .5, 1.0, and 2.0; detailed results are provided in Table S14 in Multimedia Appendix 1. Across these smoothing parameters, the personalized strategy increased mean annual effectiveness by 0.00136-0.00178 QALYs and increased mean annual cost by 19.95-53.67 CNY compared with standardized follow-up. The corresponding ICERs ranged from 14,716 to 31,151 CNY/QALY. All ICERs remained substantially below the willingness-to-pay threshold of 287,248 CNY/QALY, indicating that the projected cost-effectiveness results remained consistent across the examined transition-probability smoothing parameters.

We also performed deterministic 1-way sensitivity analyses for health utility values, complication-related medical costs, the annual baseline-management cost ceiling *c*_max_, follow-up costs, the algorithmic discount factor, and travel-related disutility. The parameter ranges are reported in Table S15 in Multimedia Appendix 1, and the corresponding results are presented in Table S16 in Multimedia Appendix 1. Across these deterministic scenarios, the personalized strategy increased annual effectiveness by 0.00124-0.00160 QALYs and increased annual cost by 21.74-55.68 CNY compared with standardized follow-up. The corresponding ICERs ranged from 14,987 to 35,613 CNY/QALY. The ICER for the personalized strategy remained below the willingness-to-pay threshold of 287,248 CNY/QALY across all examined deterministic scenarios. Varying the algorithmic discount factor only had a little effect on the conclusion: with no discounting, the personalized strategy increased annual effectiveness by 0.00145 QALYs and annual cost by 25.42 CNY, resulting in an ICER of 17,494 CNY/QALY. With a monthly factor of 0.9959, corresponding to an annual discount rate of 5%, the corresponding values were 0.00145 QALYs, 25.29 CNY, and 17,408 CNY/QALY. Both ICERs remained far below the willingness-to-pay threshold. In the willingness-to-pay–dependent policy re-optimization analyses, the MDP was re-solved using willingness-to-pay objectives corresponding to 1 and 3 times China’s 2024 per capita gross domestic product. Under the 1× gross domestic product objective, the personalized strategy increased effectiveness by 0.00140 QALYs and cost by 18.65 CNY, resulting in an ICER of 13,295 CNY/QALY. Under the 3× gross domestic product objective, the personalized strategy increased effectiveness by 0.00145 QALYs and cost by 24.67 CNY, resulting in an ICER of 17,016 CNY/QALY. Both ICERs were below their corresponding willingness-to-pay thresholds. Varying the annual baseline-management cost ceiling *c*_max_ to 12,000, 15,000, and 18,000 CNY/year produced ICERs of 14,987, 17,016, and 19,018 CNY/QALY, respectively; the 15,000 CNY/year scenario reproduced the base-case result.

### External Validation of Risk Prediction Models in the Shanghai Cohort

The final risk-prediction models developed in Nanjing were applied without reestimating model parameters in the independent Shanghai cohort of 25,506 patients from 10 communities (mean follow-up 1.87 years). The eligible incident-complication validation set contained 25,499 observations with 1742 events and yielded an external C-index of 0.778. Mortality validation used all 25,506 observations, including 125 deaths, and yielded an external C-index of 0.812. As the Shanghai cohort had a shorter follow-up duration than the Nanjing development cohort, these analyses validate discrimination rather than the MDP policy, cost-effectiveness projections, or long-term clinical effectiveness.

## Discussion

### Principal Findings

Using longitudinal primary-care data, we developed a 12-month finite-horizon MDP that selected follow-up timing and modality by maximizing expected net health benefit under prespecified utility, cost, access, and willingness-to-pay parameters. In both the example-patient and simulated-cohort analyses, the personalized policy produced small projected QALY gains at modest incremental program cost. In the 1000-patient S0 cohort, the ICER was 17,016 CNY/QALY, below the modeled willingness-to-pay threshold of 287,248 CNY/QALY. End-of-horizon complication-state occupancy was nearly identical between strategies, whereas modeled mortality was slightly lower under the personalized policy. As action-conditional transitions were estimated from observational records and may reflect confounding by indication, these differences are model-based projections and should not be interpreted as causal effects of follow-up modality.

Deterministic sensitivity analyses preserved the overall cost-effectiveness conclusion across the examined transition-smoothing, utility, cost, travel-disutility, willingness-to-pay, cycle-convention, and discount-factor scenarios. These analyses support structural robustness within the specified model but do not quantify sampling or joint parameter uncertainty; accordingly, they do not provide a probability that the personalized strategy is cost-effective. The higher modeled cost arose from both additional modality-specific follow-up charges and greater accrual of baseline management costs over the slightly larger number of expected living patient-months.

### Comparison With Prior Work

Prior diabetes follow-up research has examined encounter frequency, guideline adherence, telemedicine, personalized care systems, and self-management support [15-17,33-36]. Risk-based follow-up has also been evaluated in other chronic-disease settings [18,37,38]. These studies inform who may need more intensive care or how follow-up can be delivered, but fewer provide an explicit sequential decision framework that jointly selects timing and modality while incorporating expected health benefit, accessibility, and resource use. Related methodological work has applied machine-learning methods to predict follow-up intervals and has used MDP or approximate dynamic programming formulations to schedule health care encounters and allocate resources in other service settings [20,21,39].

Our study extends this literature by formulating follow-up planning as a sequential decision problem and incorporating QALDs and program costs directly into the MDP reward structure. The model did not project short-term cost savings; instead, it generated a small additional expected health benefit at an incremental modeled cost. This distinction is important in primary care, where decision support should make the trade-off between expected benefit and resource use explicit rather than equating personalization with lower expenditure.

The framework should be viewed as a candidate decision-support approach rather than a ready-to-deploy scheduling system. After prospective validation, it could support structured planning of follow-up frequency and modality using routinely collected clinical information together with prespecified utility, cost, and accessibility parameters. Its effects on clinician workload, patient adherence, service substitution, and implementation feasibility were not evaluated here and should be established before the model is used to modify mandated or routine follow-up schedules.

The incident-complication model was highly parsimonious: lambda.1se retained duration of diabetes alone, with apparent, optimism-corrected, and external C-indices of 0.777, 0.777, and 0.778, respectively. The lambda.min model retained 65 predictors but had lower external discrimination, and the single-year analyses yielded similar discrimination with duration of diabetes alone. Several conventional metabolic markers, including fasting blood glucose, blood pressure, and BMI, provided little incremental 1-year discrimination beyond duration of diabetes in this analysis. This parsimony reflects both the 1-year prediction horizon and the prespecified lambda.1se tuning rule, under which duration of diabetes alone achieved discrimination comparable to that of the larger lambda.min model. These findings are specific to 1-year risk discrimination in the available data and do not imply that these or other established clinical risk factors are clinically unimportant. For mortality, lambda.1se retained 7 predictors; although lambda.min yielded a higher external C-index (0.833 vs 0.812), the prespecified parsimonious model remained primary rather than selecting a model based on external performance. The 500-tree random survival forest sensitivity analysis changed external C-indices by only −0.002 for incident complications and +0.002 for mortality. Thus, the detected proportional-hazards deviations in the mortality model did not materially impair discrimination. These risk models were complementary validation analyses and supplied no numerical input to the MDP.

### Limitations

This study has several limitations. First, action-specific transition probabilities were estimated from observational follow-up records rather than randomized or prospectively assigned interventions. They may therefore reflect care-seeking behavior, clinician decision-making, access, adherence, residual baseline imbalance, and unmeasured confounding; Laplace smoothing addresses sparse cells but not confounding by indication. Most baseline differences were small, although exercise frequency and blood type had standardized mean differences of .199. Transition-cell denominators represent patient-month transitions rather than distinct independent patients, and sampling uncertainty in these probabilities was not modeled. The incremental effectiveness estimate was also small relative to its variation across smoothing scenarios. The MDP results should therefore be interpreted as projected decision-support outputs under the estimated transition structure, rather than as causal evidence for reducing guideline-based follow-up requirements. Individualization was limited to health state, travel distance, and age because the LASSO-Cox risk estimates did not condition the action-specific transitions.

Second, random-forest–based predictor imputation does not propagate imputation uncertainty in the same way as multiple imputation. In addition, the Cox models were fitted to person-year records, so patients with longer follow-up contributed more records, although cross-validation, bootstrap resampling, and robust SEs were handled at the patient level. The models were therefore used for prediction and risk stratification rather than coefficient-level causal inference. The reported bootstrap optimism correction was conditional on the LASSO-selected predictor set and therefore quantified optimism in the Cox refitting step rather than uncertainty from repeating the complete variable-selection procedure. External calibration was not assessed, so the reported C-indices establish discrimination but not calibration of absolute risk.

Third, routine public health records did not capture medication costs, productivity losses, or the full opportunity cost of provider time, facilities, consumables, and administrative support. The analysis therefore used a restricted public health program perspective. The absence of an additional charge for the first 4 outpatient visits is a program accounting convention, not evidence of zero resource use. Adding omitted resource components could change the absolute costs and ICER in either direction, depending on differential resource use. As a substantial component of the incremental cost arises from baseline-management expenditure accruing over additional living patient-months, the cost-effectiveness ratio implied by this survival channel alone is close to the baseline-management cost divided by the utility of S0; its magnitude is therefore largely determined by this prespecified cost parameter. The baseline-management cost function was also prespecified rather than estimated from individual-level expenditure data; varying its annual ceiling, *c*_max_, from 12,000 to 18,000 CNY/year assessed structural sensitivity but did not substitute for empirical cost validation.

Fourth, travel distance was represented as a linear accessibility-related disutility. This simplification may not capture nonlinear relationships or nontravel-related barriers and may favor lower-burden modalities for patients living farther from primary care. Part of the projected QALY gain therefore reflects modeled access burden rather than biomedical disease control alone.

Fifth, the MDP states were mutually exclusive and did not explicitly represent concurrent diabetes-related complications, which may underestimate the burden of multimorbidity. The 12-month horizon also captures short-term projected cost-effectiveness rather than lifetime value, as major diabetes-related complications often develop over multiple years.

Finally, the Shanghai cohort had shorter follow-up than the Nanjing development cohort and was used only to validate risk-model discrimination; neither external calibration nor the MDP policy or economic projections were validated in Shanghai. Prospective studies should evaluate implementation effectiveness, workload, adherence, modality-specific outcomes, calibration, and generalizability, while considering risk-adjusted or causal methods for estimating action-conditional transitions.

### Conclusions

This study developed a 12-month MDP framework that uses observed action-conditional transition patterns together with prespecified utility, cost, access, and willingness-to-pay parameters to generate personalized follow-up policies for patients with type 2 diabetes. In the modeled analyses, the personalized strategy produced small projected QALY gains at modest incremental program cost, and independent Shanghai data supported the discrimination of the complementary risk-prediction models. As the decision model relied on observational transition data and partly prespecified economic parameters, these results should be regarded as model-based projections requiring prospective implementation, calibration, and economic validation rather than as causal evidence for changing guideline-mandated follow-up schedules.

## Data Availability

The individual-level data are not publicly available because they contain sensitive health information derived from routine clinical practice and public health management records. Data were deidentified before analysis and used in accordance with ethics approval and institutional data-governance requirements. Reasonable requests for access to deidentified analytic data or other nonpublic analytic materials may be directed to the corresponding author and will be considered subject to institutional approval, data-use agreements, and applicable privacy regulations.

## Appendix

Multimedia Appendix 1 Additional analysis and data tables.

## Abbreviations

ICD-10: International Classification of Diseases, 10th Revision
ICER: incremental cost-effectiveness ratio
LASSO: least absolute shrinkage and selection operator
MDP: Markov decision process
QALD: quality-adjusted life day
QALY: quality-adjusted life year
