# Supplementary material for: Markov Decision Process–Based Personalized Follow-Up Planning for Type 2 Diabetes: Retrospective Cohort Study
Source: JMIR Med Inform. 2026 Sep 2;14:e88206. doi: 10.2196/88206 (PMC13583495; doi:10.2196/88206)
Supplement: Supplementary file 1 [file medinform_v14i1e88206_app1.docx]

**Appendix 1**

1. Example Patient Information

Gender: Male; Age: 60; Travel distance: 2.0 km; Education level: High school; Occupation: Professional technician; Disability: No; Marital status: Married; Medical payment: Urban employee medical insurance; Blood type: A; Rh blood: Negative; Waist circumference: 90cm; BMI: 27.68kg/m²; Diastolic blood pressure: 85; Systolic blood pressure: 135; Body temperature: 36.7℃; Heart rate: 75 beats/minute; Genetic history: None; Drug allergy: None; Exposure history: None; Surgical history: None; Blood transfusion history: None; Family history of diabetes: Yes; Complications: None; Dietary habits: Normal; Exercise frequency: Once to three times per week; Exercise duration per session: Less than half an hour per session; Smoking: Yes; Alcohol consumption: Yes; Daily alcohol consumption: Less than 250ml; Lips: Normal; Teeth: Abnormal; Pharynx: Normal; Hearing: Normal; Motor function: Normal; Fundus: Normal; Skin: Normal; Sclera: Normal; Lymph nodes: Normal; Barrel chest: Normal; Respiratory sounds: Normal; Rale: Normal; Heart rhythm: Normal; Murmur: Normal; Tenderness: Normal; Lower limb edema: Normal; Dorsalis pedis artery: Normal; Urine protein: Normal; Urine glucose: Abnormal; Urine ketone bodies: Normal; Urine occult blood: Normal; Fecal occult blood: Normal; Electrocardiogram: Normal; Chest X-ray: Normal; Abdominal ultrasound: Normal; Cerebrovascular disease: Normal; Kidney disease: Normal; Heart disease: Normal; Vascular disease: Normal; Eye disease: Normal; Neurological disease: Normal; Hemoglobin: 129; White blood cells: 11.18; Platelets: 163; Urine microalbumin: 18mg/24h; fasting blood glucose: 7.2mmol/L; serum alanine aminotransferase (ALT): 22.4; serum aspartate aminotransferase (AST): 25.8; albumin: 45.2; total bilirubin: 11.7; conjugated bilirubin: 3.4; serum creatinine: 78.4μmol/L; blood urea nitrogen (BUN): 5.20mmol/L; serum potassium: 3.71; serum sodium: 140.5; total cholesterol: 5.89; triglycerides: 2.3; low-density lipoprotein cholesterol (LDL-C): 3.83; high-density lipoprotein cholesterol (HDL-C): 1.4.

Appendix Table 1. Complete Month-by-Month MDP-Generated Follow-Up Plans for Example Patients by Initial Health State

| State | Number of Follow-ups | Types of Follow-ups | | | | | | | | | | | |
| --- | --- | --- | --- | --- | --- | --- | --- | --- | --- | --- | --- | --- | --- |
|  |  | Month 1 | Month 2 | Month 3 | Month 4 | Month 5 | Month 6 | Month 7 | Month 8 | Month 9 | Month 10 | Month 11 | Month 12 |
| S0 | 5 |  |  |  |  | O | O | O | T |  | O |  |  |
| S1 | 8 |  | O | O |  | O | O | O | O | O |  | O |  |
| S2 | 6 |  |  | O |  | O | O | O |  | O |  | O |  |
| S3 | 6 |  |  |  | O | O |  |  | O | O | O | O |  |
| S4 | 6 |  | O |  | O | O |  | O |  | O |  | C |  |
| Note: “O” indicates outpatient follow-up, “T” indicates telephone follow-up, and “C” indicates community follow-up. States are defined as T2DM without recorded complications (S0), diabetic nephropathy (S1), diabetic retinopathy (S2), diabetic peripheral neuropathy (S3), lower-extremity arterial disease and diabetic foot (S4), and death (S5). | | | | | | | | | | | | | |

Appendix Table 2. Annual QALDs and Relative QALD Gains for the Example Patient Under Different Follow-up Plans

| State | Effectiveness (QALDs, Days) | | Difference in Effectiveness  (QALDs, Days) | Relative QALD Gain, % |
| --- | --- | --- | --- | --- |
|  | Personalized Follow-up Plan | Standardized Follow-up Plan |  |  |
| S0 | 314.12 | 313.64 | 0.48 | 0.15% |
| S1 | 272.49 | 270.88 | 1.61 | 0.59% |
| S2 | 300.15 | 298.12 | 2.03 | 0.68% |
| S3 | 278.30 | 277.51 | 0.79 | 0.29% |
| S4 | 250.31 | 249.68 | 0.63 | 0.25% |
| Note: Effectiveness was measured as annual QALDs. Relative QALD gain was calculated as the difference in QALDs divided by QALDs under the standardized follow-up plan. Differences were calculated using unrounded model outputs; displayed values may not sum exactly because of rounding. | | | | |

Appendix Table 3. Annual Costs, QALYs, and Incremental Cost-Effectiveness Ratios for the Example Patient Under Different Follow-up Plans

| State | Cost (CNY) | | Difference in Cost (CNY) | ICER  (CNY/QALY) | Supplementary ICER, (CNY/QALD) |
| --- | --- | --- | --- | --- | --- |
|  | Personalized Follow-up Plan | Standardized Follow-up Plan |  |  |  |
| S0 | 15,070.94 | 15,037.48 | 33.46 | 25,499 | 69.86 |
| S1 | 26,794.30 | 26,581.46 | 212.84 | 48,295 | 132.32 |
| S2 | 19,314.15 | 19,157.30 | 156.85 | 28,134 | 77.08 |
| S3 | 20,320.97 | 20,236.81 | 84.16 | 38,782 | 106.25 |
| S4 | 22,816.07 | 22,705.99 | 110.08 | 63,754 | 174.67 |
| Note: Costs are annual costs in CNY. QALYs were calculated by dividing QALDs by 365. ICERs were reported primarily as CNY/QALY, with CNY/QALD provided as a supplementary equivalent. ICERs were calculated using unrounded model outputs; displayed values may not sum exactly because of rounding. Annual costs represent the accumulated sum of monthly state costs and monthly follow-up costs over the 12-month horizon. | | | | | |

Appendix Table 4. Monte Carlo Algorithmic Check Results for Example Patients

| State | Strategy | Mean QALDs | Mean Cost, CNY | Final complication-state probability | Final death probability |
| --- | --- | --- | --- | --- | --- |
| S0 | Personalized | 314.26 | 15,080.33 | 5.19% | 1.28% |
| S0 | Standardized | 313.75 | 15,045.06 | 5.09% | 1.61% |
| S1 | Personalized | 272.64 | 26,808.83 | 97.80% | 2.20% |
| S1 | Standardized | 270.95 | 26,588.13 | 96.13% | 3.87% |
| S2 | Personalized | 300.40 | 19,330.20 | 97.46% | 2.54% |
| S2 | Standardized | 298.33 | 19,170.80 | 95.71% | 4.29% |
| S3 | Personalized | 278.46 | 20,332.70 | 97.90% | 2.10% |
| S3 | Standardized | 277.63 | 20,245.66 | 96.73% | 3.27% |
| S4 | Personalized | 250.45 | 22,828.69 | 97.70% | 2.30% |
| S4 | Standardized | 249.75 | 22,712.42 | 96.67% | 3.33% |
| Note: Results were based on 10,000 Monte Carlo simulated trajectories. Final complication-state probability refers to the probability of occupying S1-S4 at the end of the 12-month horizon, rather than cumulative incident complications. For patients initialized in S1-S4, final complication-state probability includes remaining in the baseline complication state and should not be interpreted as incident complication risk. | | | | | |

**Appendix 2**

Appendix Table 5. Group-Level Annual QALYs, Costs, and Incremental Cost-Effectiveness Ratios in the Specified Simulated Cohort

| Outcome | Personalized | Standardized | Difference |
| --- | --- | --- | --- |
| Mean annual QALDs, days | 314.05 | 313.52 | 0.529 |
| Mean annual QALYs | 0.86041 | 0.85896 | 0.00145 |
| Mean annual cost, CNY | 15,032.27 | 15,007.60 | 24.67 |
| ICER, CNY/QALY | — | — | 17,016 |
| Supplementary ICER, CNY/QALD | — | — | 46.62 |
| Note: The simulated cohort was initialized in S0 with respect to the Markov decision process health state but was not composed of 1,000 identical replications of the illustrative patient. The simulated cohort consisted of 1,000 heterogeneous patients initialized in S0, with variation in age and travel distance. The base utility of S0 was fixed at 0.868, while age, travel distance, and action-specific travel disutility contributed to heterogeneity in simulated outcomes. All patients used the same fixed monthly cycle duration of 365/12 days. Cohort-level mean QALDs were calculated by summing monthly QALDs over all 12 cycles and averaging across the 1,000 patients. No additional penalty term or duplicate utility adjustment was applied in the group-level simulation. QALYs were calculated by dividing QALDs by 365. Difference was defined as the personalized follow-up strategy minus the standardized follow-up strategy. Differences and ICERs were calculated using unrounded outputs; displayed values may therefore not reproduce them exactly because of rounding. ICERs were reported primarily as CNY/QALY, with CNY/QALD provided as a supplementary equivalent. Annual costs were obtained by summing monthly costs over the 12-month horizon; annual baseline and complication-related cost parameters were converted to monthly costs by dividing by 12 before entering each monthly MDP cycle. | | | |

Appendix Table 6. Decomposition of Group-Level Annual Costs

| Cost component | Personalized | Standardized | Difference |
| --- | --- | --- | --- |
| Baseline management cost, CNY | 14,848.24 | 14,832.59 | 15.64 |
| Complication-related cost, CNY | 169.09 | 175.00 | −5.91 |
| Follow-up cost, CNY | 14.94 | 0.00 | 14.94 |
| Total annual cost, CNY | 15,032.27 | 15,007.60 | 24.67 |
| Living patient-months | 11.9320 | 11.9195 | 0.0126 |
| Note: Difference was calculated as the personalized strategy minus standardized follow-up. Differences were calculated using unrounded outputs; displayed values may therefore not reproduce them exactly because of rounding. The total incremental cost of 24.67 CNY was the sum of a 15.64 CNY increase in baseline diabetes-management expenditure, a 5.91 CNY reduction in complication-related cost, and a 14.94 CNY increase in additional modality-specific follow-up charges. The greater baseline expenditure under the personalized strategy was consistent with its 0.0126 additional expected living patient-months. Under the specified accounting convention, the first four outpatient visits were assigned no additional modality-specific charge; therefore, the value of 0.00 CNY for standardized follow-up should not be interpreted as indicating zero provider resource use. The follow-up cost of 14.94 CNY comprises 13.29 CNY for telephone follow-up (0.9633 expected contacts at 13.8 CNY), 0.42 CNY for community follow-up (0.0105 contacts at 40 CNY), and 1.23 CNY for outpatient visits from the fifth onward (0.1022 expected charged visits at 12 CNY); the first four outpatient visits carried no additional modality-specific charge. Outpatient visits beyond the fourth arise in trajectories that enter a complication state, in which the optimized policy prescribes more frequent outpatient follow-up, so the cohort mean of 1.74 outpatient contacts is compatible with a small expected number of charged visits. Values are displayed rounded (unrounded components: 1.2260, 13.2936 and 0.4214 CNY). | | | |

Appendix Table 7. Reconciliation of Group-Level Annual QALD Accumulation

| Strategy | Sum of patient annual QALDs | Mean from patient-level sum | Sum of monthly QALDs | Mean from monthly sum | Reported mean | Reconciled |
| --- | --- | --- | --- | --- | --- | --- |
| Personalized | 314,048.3468 | 314.0483 | 314,048.3468 | 314.0483 | 314.0483 | Yes |
| Standardized | 313,519.1270 | 313.5191 | 313,519.1270 | 313.5191 | 313.5191 | Yes |

Appendix Table 8. Worked Month-by-Month Expected QALD and Cost Accumulation for a Selected Simulated Patient

| Month | Cycle days | Alive prob. | Adj. utility | Monthly QALDs | Baseline cost | Comp. cost | F/U cost | Total cost | Cum. QALDs | Cum. cost |
| --- | --- | --- | --- | --- | --- | --- | --- | --- | --- | --- |
| 1 | 30.4167 | 1.0000 | 0.8680 | 26.4017 | 1,248.12 | 0.00 | 0.00 | 1,248.12 | 26.4017 | 1,248.12 |
| 2 | 30.4167 | 0.9995 | 0.8671 | 26.3754 | 1,247.54 | 2.52 | 0.00 | 1,250.07 | 52.7771 | 2,498.19 |
| 3 | 30.4167 | 0.9988 | 0.8661 | 26.3439 | 1,246.67 | 4.85 | 0.00 | 1,251.51 | 79.1209 | 3,749.70 |
| 4 | 30.4167 | 0.9978 | 0.8647 | 26.3020 | 1,245.34 | 7.30 | 0.00 | 1,252.63 | 105.4229 | 5,002.34 |
| 5 | 30.4167 | 0.9965 | 0.8631 | 26.2534 | 1,243.72 | 9.91 | 0.00 | 1,253.63 | 131.6763 | 6,255.97 |
| 6 | 30.4167 | 0.9952 | 0.8608 | 26.1831 | 1,242.15 | 12.37 | 0.00 | 1,254.52 | 157.8594 | 7,510.49 |
| 7 | 30.4167 | 0.9942 | 0.8594 | 26.1392 | 1,240.88 | 15.54 | 0.01 | 1,256.43 | 183.9986 | 8,766.92 |
| 8 | 30.4167 | 0.9934 | 0.8589 | 26.1245 | 1,239.83 | 18.47 | 13.31 | 1,271.61 | 210.1230 | 10,038.52 |
| 9 | 30.4167 | 0.9926 | 0.8570 | 26.0660 | 1,238.91 | 21.27 | 0.07 | 1,260.24 | 236.1890 | 11,298.76 |
| 10 | 30.4167 | 0.9910 | 0.8551 | 26.0106 | 1,236.94 | 23.83 | 0.08 | 1,260.85 | 262.1996 | 12,559.61 |
| 11 | 30.4167 | 0.9900 | 0.8545 | 25.9924 | 1,235.65 | 26.31 | 0.72 | 1,262.69 | 288.1920 | 13,822.30 |
| 12 | 30.4167 | 0.9873 | 0.8518 | 25.9083 | 1,232.25 | 28.68 | 0.00 | 1,260.93 | 314.1003 | 15,083.23 |
| Note: The table presents a worked expected-value trace for one selected simulated patient under the MDP-based personalized follow-up strategy. Because outcomes were calculated using probability propagation across health states, the monthly values represent probability-weighted expectations rather than a single realized trajectory. Each monthly cycle had a fixed duration of 365/12 = 30.4167 days. Expected monthly QALDs were calculated as the fixed cycle duration multiplied by expected adjusted utility. Annual QALDs and annual costs equal the sums of the corresponding 12 monthly values. Values are displayed in rounded form; reconciliation was performed using unrounded outputs. | | | | | | | | | | |

Appendix Table 9. Reconciliation of Monthly and Annual Outcomes for Three Worked Patient Examples Under Both Strategies

| Pt. | Strategy | Monthly QALDs sum | Baseline cost | Comp. cost | F/U cost | Monthly cost sum | Reported QALDs | Reported cost | QALD OK | Cost OK |
| --- | --- | --- | --- | --- | --- | --- | --- | --- | --- | --- |
| 1 | Personalized | 314.1003 | 14,897.99 | 171.05 | 14.19 | 15,083.23 | 314.1003 | 15,083.23 | Yes | Yes |
| 1 | Standardized | 313.6250 | 14,876.95 | 175.00 | 0.00 | 15,051.95 | 313.6250 | 15,051.95 | Yes | Yes |
| 2 | Personalized | 314.0814 | 14,819.16 | 170.99 | 14.10 | 15,004.25 | 314.0814 | 15,004.25 | Yes | Yes |
| 2 | Standardized | 313.6078 | 14,799.60 | 175.00 | 0.00 | 14,974.60 | 313.6078 | 14,974.60 | Yes | Yes |
| 3 | Personalized | 314.0774 | 14,913.47 | 170.98 | 14.04 | 15,098.50 | 314.0774 | 15,098.50 | Yes | Yes |
| 3 | Standardized | 313.6026 | 14,893.80 | 175.00 | 0.00 | 15,068.80 | 313.6026 | 15,068.80 | Yes | Yes |
| Note: Monthly QALDs and cost components were summed over all 12 cycles and compared directly with the independently reported annual patient-level outcomes. All six patient-strategy combinations reconciled exactly when calculated using unrounded values. Displayed values are rounded. | | | | | | | | | | |

Appendix Table 10. Cohort-Mean Month-by-Month QALD and Cost Accumulation Under Both Strategies

| Month | Cycle days | Pers. monthly QALDs | Pers. cumulative QALDs | Pers. monthly cost | Pers. cumulative cost | Std. monthly QALDs | Std. cumulative QALDs | Std. monthly cost | Std. cumulative cost |
| --- | --- | --- | --- | --- | --- | --- | --- | --- | --- |
| 1 | 30.4167 | 26.4017 | 26.4017 | 1,244.40 | 1,244.40 | 26.3509 | 26.3509 | 1,244.40 | 1,244.40 |
| 2 | 30.4167 | 26.3754 | 52.7770 | 1,246.35 | 2,490.75 | 26.3557 | 52.7066 | 1,246.48 | 2,490.88 |
| 3 | 30.4167 | 26.3438 | 79.1208 | 1,247.79 | 3,738.54 | 26.3242 | 79.0308 | 1,247.92 | 3,738.80 |
| 4 | 30.4167 | 26.3021 | 105.4229 | 1,248.92 | 4,987.46 | 26.2318 | 105.2626 | 1,249.04 | 4,987.84 |
| 5 | 30.4167 | 26.2509 | 131.6738 | 1,249.92 | 6,237.38 | 26.2276 | 131.4902 | 1,249.84 | 6,237.68 |
| 6 | 30.4167 | 26.1958 | 157.8696 | 1,250.91 | 7,488.29 | 26.1804 | 157.6706 | 1,250.70 | 7,488.38 |
| 7 | 30.4167 | 26.1421 | 184.0116 | 1,252.21 | 8,740.50 | 26.0757 | 183.7463 | 1,251.42 | 8,739.80 |
| 8 | 30.4167 | 26.1143 | 210.1259 | 1,266.95 | 10,007.45 | 26.0877 | 209.8340 | 1,253.26 | 9,993.06 |
| 9 | 30.4167 | 26.0762 | 236.2022 | 1,255.58 | 11,263.03 | 26.0279 | 235.8619 | 1,253.47 | 11,246.53 |
| 10 | 30.4167 | 26.0076 | 262.2098 | 1,256.74 | 12,519.77 | 25.9088 | 261.7707 | 1,253.33 | 12,499.86 |
| 11 | 30.4167 | 25.9620 | 288.1717 | 1,257.07 | 13,776.84 | 25.9181 | 287.6888 | 1,254.51 | 13,754.37 |
| 12 | 30.4167 | 25.8766 | 314.0483 | 1,255.43 | 15,032.27 | 25.8303 | 313.5191 | 1,253.23 | 15,007.60 |
| Total | 365.0000 | 314.0483 | — | 15,032.27 | — | 313.5191 | — | 15,007.60 | — |
| Note: Values are cohort means across the 1,000 simulated patients and were computed in the same pass that produced **Appendix Tables 5, 5A, 7 and 8.** Each monthly value is the mean across patients of the expected QALDs (or cost) accrued in that cycle under probability propagation across health states; each monthly cycle has a fixed duration of 365/12 = 30.4167 days. The twelve monthly values sum exactly to the annual cohort means reported in Appendix Tables 5 and 6. The cohort-mean survival probabilities underlying these values sum to 11.9320 and 11.9195 living patient-months under the personalized and standardized strategies respectively, equal to the values reported in Appendix Table 6. Under the standardized strategy an outpatient follow-up is scheduled in months 1, 4, 7 and 10, which is reflected in the corresponding monthly values through the travel-related disutility term. Values are displayed rounded and cumulative values were computed from unrounded outputs; reconciliation was performed using unrounded outputs throughout. Appendix Table 8 reports the corresponding trace for a single selected patient and is provided as an illustrative worked example. | | | | | | | | | |

Appendix Table 11. Group-level Strategy Summary for the Specified Simulated Cohort Initialized in S0

| Initial state | Strategy | Mean annual QALDs | Mean annual cost | Mean total follow-ups | Outpatient | Telephone | Community |
| --- | --- | --- | --- | --- | --- | --- | --- |
| S0 | Personalized | 314.05 | 15,032.27 | 2.72 | 1.74 | 0.96 | 0.01 |
| S0 | Standardized | 313.52 | 15,007.60 | 3.98 | 3.98 | 0.00 | 0.00 |
| Note: The simulated cohort consisted of 1,000 heterogeneous patients initialized in S0, with variation in age and travel distance. The base utility of S0 was fixed at 0.868 for all patients. Follow-up counts are expected counts calculated by weighting monthly actions by the corresponding state-occupancy probabilities; therefore, they need not be integers. All QALDs were accumulated over 12 fixed monthly cycles of 365/12 days. Expected follow-up counts are displayed rounded to two decimal places; component counts may not sum exactly to the displayed total (unrounded: 1.7425 + 0.9633 + 0.0105 = 2.7163). | | | | | | | |

Appendix Table 12. Monthly Complication-state Counts and Proportions Under Different Strategies

| Month | Personalized Count | Standardized Count | Difference | Personalized Proportion | Standardized Proportion | Difference |
| --- | --- | --- | --- | --- | --- | --- |
| 1 | 4.12 | 5.45 | -1.33 | 0.41% | 0.55% | -0.13% |
| 2 | 8.02 | 9.34 | -1.32 | 0.80% | 0.93% | -0.13% |
| 3 | 12.08 | 13.39 | -1.31 | 1.21% | 1.34% | -0.13% |
| 4 | 16.33 | 17.86 | -1.53 | 1.63% | 1.79% | -0.15% |
| 5 | 20.43 | 21.83 | -1.40 | 2.04% | 2.18% | -0.14% |
| 6 | 25.13 | 26.08 | -0.96 | 2.51% | 2.61% | -0.10% |
| 7 | 29.60 | 30.69 | -1.09 | 2.96% | 3.07% | -0.11% |
| 8 | 34.14 | 34.66 | -0.52 | 3.41% | 3.47% | -0.05% |
| 9 | 38.28 | 38.62 | -0.34 | 3.83% | 3.86% | -0.03% |
| 10 | 42.49 | 42.61 | -0.12 | 4.25% | 4.26% | -0.01% |
| 11 | 46.34 | 46.36 | -0.02 | 4.63% | 4.64% | 0.00% |
| 12 | 51.14 | 51.15 | -0.01 | 5.114% | 5.115% | -0.001% |
| Note: Values are expected counts and proportions in the simulated cohort of 1,000 patients. Complication-state count refers to the expected number of patients occupying states S1–S4 at the end of each monthly cycle. Difference was defined as the personalized follow-up strategy minus the standardized follow-up strategy. Differences were calculated using unrounded model outputs and may therefore not equal the difference between the displayed rounded values. | | | | | | |

Appendix Table 13. Monthly Death Counts and Cumulative Mortality Proportions Under Different Strategies

| Month | Personalized Count | Standardized Count | Difference | Personalized Proportion | Standardized Proportion | Difference |
| --- | --- | --- | --- | --- | --- | --- |
| 1 | 0.46 | 1.03 | -0.57 | 0.05% | 0.10% | -0.06% |
| 2 | 1.17 | 1.73 | -0.57 | 0.12% | 0.17% | -0.06% |
| 3 | 2.23 | 2.80 | -0.57 | 0.22% | 0.28% | -0.06% |
| 4 | 3.53 | 4.30 | -0.78 | 0.35% | 0.43% | -0.08% |
| 5 | 4.75 | 5.58 | -0.84 | 0.47% | 0.56% | -0.08% |
| 6 | 6.02 | 7.09 | -1.08 | 0.60% | 0.71% | -0.11% |
| 7 | 7.09 | 7.95 | -0.85 | 0.71% | 0.79% | -0.09% |
| 8 | 7.84 | 9.72 | -1.88 | 0.78% | 0.97% | -0.19% |
| 9 | 9.73 | 11.80 | -2.07 | 0.97% | 1.18% | -0.21% |
| 10 | 11.19 | 12.84 | -1.64 | 1.12% | 1.28% | -0.16% |
| 11 | 13.96 | 15.70 | -1.74 | 1.40% | 1.57% | -0.17% |
| 12 | 14.37 | 16.10 | -1.74 | 1.437% | 1.610% | -0.173% |
| Note: Values are expected counts and proportions in the simulated cohort of 1,000 patients. Because death was modeled as an absorbing state, the death proportion at each month represents cumulative expected mortality by the end of that month. Difference was defined as the personalized follow-up strategy minus the standardized follow-up strategy. Differences were calculated using unrounded model outputs and may therefore not equal the difference between the displayed rounded values. | | | | | | |

Expected living patient-months were calculated from the probabilities of being alive at the beginning of each monthly cycle. Specifically, the month 0 through month 11 survival probabilities were summed. This calculation yielded 11.9320 expected living patient-months under the personalized strategy and 11.9195 under the standardized strategy, consistent with the independently reported results in Appendix Table 6.

$$M_{\text{alive}}^{\pi}=\sum_{t=1}^{12} \left( 1-p_{D,t-1}^{\pi} \right), p_{D,0}^{\pi}=0$$

$$M_{\text{alive}}^{\text{personalized}}=11.9320248\approx11.9320$$

$$M_{\text{alive}}^{\text{standardized}}=11.9194548\approx11.9195$$

$$\Delta M_{\text{alive}}=11.9320248-11.9194548=0.0125700\approx0.0126$$

Appendix Table 14. Sensitivity Analysis of Group-Level QALYs, Costs, and ICERs Using Alternative Laplace Smoothing Parameters

| Alpha | Mean QALDs, personalized | Mean QALDs, standardized | ΔQALDs | ΔQALYs | Mean cost, personalized | Mean cost, standardized | ΔCost | ICER, CNY/QALY | ICER, CNY/QALD |
| --- | --- | --- | --- | --- | --- | --- | --- | --- | --- |
| 0.1 | 314.22 | 313.57 | 0.650 | 0.00178 | 15,060.57 | 15,006.90 | 53.67 | 30,138 | 82.57 |
| 0.5 | 314.14 | 313.55 | 0.595 | 0.00163 | 15,058.02 | 15,007.21 | 50.80 | 31,151 | 85.34 |
| 1.0 | 314.05 | 313.52 | 0.529 | 0.00145 | 15,032.27 | 15,007.60 | 24.67 | 17,016 | 46.62 |
| 2.0 | 313.95 | 313.46 | 0.495 | 0.00136 | 15,028.29 | 15,008.34 | 19.95 | 14,716 | 40.32 |
| Note: Alpha denotes the Laplace smoothing parameter used in transition-probability estimation. QALYs were calculated by dividing QALDs by 365. Differences were calculated as the MDP-based personalized strategy minus standardized follow-up. ICERs were calculated using unrounded model outputs and reported primarily as CNY/QALY, with CNY/QALD reported as a supplementary equivalent. Displayed values may not reproduce the ICER exactly because of rounding. All ICERs were below the willingness-to-pay threshold of 287,248 CNY/QALY. The smoothed transition probability for an admissible next state was calculated as (n + alpha) / (denominator + alpha x k), where n is the observed transition count, the denominator is the total number of observed transitions from that state under that action in that month, and k is the number of admissible next states; inadmissible transitions were assigned zero probability. The transition table provided for peer review reports n, the denominator, and k for every cell, so every row of this table can be recomputed from that file alone, without any individual-level transition record. The transition structure comprised 288 month-by-action-by-state cells. Four cells had no observed transitions, all in the absorbing death state S5 in month 12, where only one next state is admissible and the smoothed probability therefore remained 1. Among the 240 cells starting from a living state, the smallest denominator was 28 and the median was 1,453; only 1 of these cells had a denominator below 30 and 24 had a denominator below 100, and telephone follow-up was the sparsest action, with a median denominator of 131.5. Across all 288 cells, including those starting from the absorbing death state, 17 cells had a denominator below 30 and 55 had a denominator below 100. | | | | | | | | | |

Appendix Table 15. Parameters Used in Deterministic Sensitivity and Willingness-to-Pay–Dependent Policy Re-Optimization Analyses

| Parameter group | Parameter | Base-case value | Lower bound | Upper bound | Unit | Variation |
| --- | --- | --- | --- | --- | --- | --- |
| Health utility values | Utility of S0 no complication | 0.868 | 0.694 | 1.000 | utility | ±20%; upper bound capped at 1.0 |
| Health utility values | Utility of S1 kidney | 0.753 | 0.602 | 0.904 | utility | ±20%; upper bound capped at 1.0 |
| Health utility values | Utility of S2 retinopathy | 0.830 | 0.664 | 0.996 | utility | ±20%; upper bound capped at 1.0 |
| Health utility values | Utility of S3 neuropathy | 0.769 | 0.615 | 0.923 | utility | ±20%; upper bound capped at 1.0 |
| Health utility values | Utility of S4 lower limb foot | 0.692 | 0.554 | 0.830 | utility | ±20%; upper bound capped at 1.0 |
| Complication-related medical costs | Annual medical cost of S1 kidney | 12,000.00 | 9,600.00 | 14,400.00 | CNY/year | ±20% |
| Complication-related medical costs | Annual medical cost of S2 retinopathy | 4,500.00 | 3,600.00 | 5,400.00 | CNY/year | ±20% |
| Complication-related medical costs | Annual medical cost of S3 neuropathy | 5,500.00 | 4,400.00 | 6,600.00 | CNY/year | ±20% |
| Complication-related medical costs | Annual medical cost of S4 lower limb foot | 8,000.00 | 6,400.00 | 9,600.00 | CNY/year | ±20% |
| Follow-up costs | Outpatient follow-up cost | 12.00 | 9.60 | 14.40 | CNY/visit | ±20% |
| Follow-up costs | Telephone follow-up cost | 13.80 | 11.04 | 16.56 | CNY/call | ±20% |
| Follow-up costs | Community follow-up cost | 40.00 | 32.00 | 48.00 | CNY/visit | ±20% |
| Travel-related disutility | Travel disutility coefficient | 0.00032 | 0.000256 | 0.000384 | utility loss per km | ±20% |
| Baseline management cost | Baseline-management cost ceiling c_max_ | 15,000 | 12,000 | 18,000 | CNY/year | ±20%; policy re-optimized |
| WTP-dependent policy objective | WTP used in net health benefit reward | 287,248 | 95,749 | 287,248 | CNY/QALY | 1× and 3× China’s 2024 per capita GDP per QALY |
| Algorithmic discount factor | Monthly discount factor in the Bellman recursion | 0.98 | — | — | per month | Alternative scenarios: 0.9959; 1.000 |
| Note: Health utility values, complication-related medical costs, follow-up costs, and travel-related disutility were varied by ±20% from their base-case values. The travel-related disutility coefficient was varied from the base-case value of 0.00032 utility loss per km to 0.000256 and 0.000384. The upper bound of health utility values was capped at 1.0. The willingness-to-pay parameter was examined at 1× and 3× China’s 2024 per capita GDP per QALY as part of WTP-dependent policy re-optimization because it entered the MDP reward function through the net health benefit objective. Annual medical cost parameters were converted to monthly costs by dividing by 12 in the MDP; follow-up costs were accrued only in the month in which the corresponding follow-up action occurred. Follow-up cost parameters f_1_, f_2_ and f_3_ denote the additional outpatient follow-up charge applied from the fifth outpatient visit onward, the modeled telephone follow-up charge per call, and the modeled community follow-up charge per visit, respectively, and are distinct from the annual baseline management cost c_base,i_ and the state-specific annual complication-related cost parameters c_comp,s_. The algorithmic discount factor was additionally varied because it enters the Bellman recursion and therefore affects the optimized policy, although it does not enter the reporting of annual QALDs and costs, which are undiscounted sums of the 12 monthly values. A monthly factor of 1.000 corresponds to no discounting and 0.9959 to an annual discount rate of 5%. The annual baseline-management cost ceiling c_max_ was varied to 12,000 and 18,000 CNY/year around its unchanged base-case value of 15,000 CNY/year, and the policy was re-optimized in each scenario. | | | | | | |

Appendix Table 16. Deterministic Sensitivity and Willingness-to-Pay–Dependent Policy Re-Optimization Results for Group-Level Cost-Effectiveness

| Scenario | Level | ΔQALDs | ΔQALYs | ΔCost, CNY | ICER, CNY/QALY | ICER, CNY/QALD |
| --- | --- | --- | --- | --- | --- | --- |
| Base case | Base | 0.529 | 0.00145 | 24.67 | 17,016 | 46.62 |
| Health utility values | Low | 0.453 | 0.00124 | 21.77 | 17,554 | 48.09 |
| Health utility values | High | 0.583 | 0.00160 | 27.91 | 17,468 | 47.86 |
| Complication-related medical costs | Low | 0.529 | 0.00145 | 25.97 | 17,902 | 49.05 |
| Complication-related medical costs | High | 0.529 | 0.00145 | 23.29 | 16,072 | 44.03 |
| Outpatient follow-up cost | Low | 0.530 | 0.00145 | 25.13 | 17,299 | 47.39 |
| Outpatient follow-up cost | High | 0.529 | 0.00145 | 24.34 | 16,811 | 46.06 |
| Telephone follow-up cost | Low | 0.529 | 0.00145 | 22.01 | 15,183 | 41.60 |
| Telephone follow-up cost | High | 0.529 | 0.00145 | 27.33 | 18,850 | 51.64 |
| Community follow-up cost | Low | 0.571 | 0.00156 | 55.68 | 35,613 | 97.57 |
| Community follow-up cost | High | 0.529 | 0.00145 | 24.76 | 17,074 | 46.78 |
| Baseline-management cost ceiling c_max_ (CNY/year) | 12,000 | 0.530 | 0.00145 | 21.74 | 14,987 | 41.06 |
| Baseline-management cost ceiling c_max_ (CNY/year) | 15,000 (base) | 0.529 | 0.00145 | 24.67 | 17,016 | 46.62 |
| Baseline-management cost ceiling c_max_ (CNY/year) | 18,000 | 0.529 | 0.00145 | 27.55 | 19,018 | 52.10 |
| Travel disutility | Low | 0.504 | 0.00138 | 27.15 | 19,666 | 53.88 |
| Travel disutility | High | 0.559 | 0.00153 | 23.06 | 15,048 | 41.23 |
| WTP-dependent policy re-optimization | 1×GDP | 0.512 | 0.00140 | 18.65 | 13,295 | 36.42 |
| WTP-dependent policy re-optimization | 3×GDP | 0.529 | 0.00145 | 24.67 | 17,016 | 46.62 |
| Discount factor | 1.000 (undiscounted) | 0.530 | 0.00145 | 25.42 | 17,494 | 47.93 |
| Discount factor | 0.9959 (5% per year) | 0.530 | 0.00145 | 25.29 | 17,408 | 47.69 |
| Note: In all deterministic sensitivity and willingness-to-pay scenarios, the MDP policy was re-optimized under the scenario-specific parameter values; therefore, changes in unit costs may alter the selected follow-up mix rather than only scaling the cost of a fixed strategy. ΔQALDs, ΔQALYs, and ΔCost were calculated as the values under the MDP-based personalized strategy minus those under standardized follow-up. QALYs were calculated by dividing QALDs by 365. ICERs were calculated using unrounded model outputs. Displayed values may therefore not reproduce the ICER exactly because of rounding. A scenario was considered cost-effective when its ICER was below the corresponding willingness-to-pay threshold. The WTP-dependent policy re-optimization rows represent scenarios in which the MDP was re-solved using alternative WTP parameters; they should not be interpreted as fixed-policy acceptance-threshold analyses. The discount factor rows re-solved the Markov decision process using alternative values of the algorithmic discount factor applied to the expected future value in the Bellman recursion. A monthly factor of 1.000 corresponds to no discounting, and 0.9959 corresponds to an annual discount rate of 5%, the rate recommended by the China Guidelines for Pharmacoeconomic Evaluations; the base-case value of 0.98 corresponds to a higher effective annual rate. Because the discount factor enters only the policy optimization and not the reporting of annual QALDs and costs, these rows reflect changes in the optimized follow-up policy. The cmax rows re-solved the policy at 12,000, 15,000, and 18,000 CNY/year; the 15,000 CNY/year row reproduces the unchanged base case. | | | | | | |

**Appendix 3**

Appendix Table 17. Sensitivity Analysis Using Random Survival Forest Models Not Requiring the Proportional Hazards Assumption

| Outcome | Model | Development C-index | Conditional patient-level bootstrap-corrected C-index | External C-index | Difference in external C-index from LASSO-Cox |
| --- | --- | --- | --- | --- | --- |
| Incident complication | LASSO-Cox refit | 0.777 | 0.777 | 0.778 | Reference |
| Incident complication | Random survival forest | 0.781 | - | 0.776 | −0.002 |
| Mortality | LASSO-Cox refit | 0.768 | 0.767 | 0.812 | Reference |
| Mortality | Random survival forest | 0.791 | - | 0.814 | +0.002 |
| Note: Random survival forest models were used only as sensitivity analyses to assess whether relaxing the proportional hazards assumption materially affected discrimination, particularly for mortality, for which scaled Schoenfeld residual testing indicated significant proportional-hazards deviations. No evidence of proportional-hazards violation was detected for the incident-complication model (global P=.927). Each forest used 500 trees and the same LASSO-selected predictor set as the corresponding primary Cox model. The LASSO-Cox models were retained as the primary risk models because they provide a parsimonious and interpretable set of predictors. Development C-index values are apparent, in-sample values and were not corrected for optimism; they are therefore not directly comparable across model classes, and the external C-index is the informative comparison. In external validation, the random survival forest showed a slightly lower C-index for incident complications (difference −0.002) and a slightly higher C-index for mortality (difference +0.002); neither difference represented a material improvement in discrimination. | | | | | |

**Appendix 4**

Appendix Table 18. Missingness of Core Variables Used for Risk Prediction, State Identification, and MDP Parameterization

| Variable | Role in analysis | Missing count | Missing proportion | Handling |
| --- | --- | --- | --- | --- |
| Age | Cox/MDP Input | - | - | Complete |
| Sex | Cox Candidate Predictor | - | - | Complete |
| Fasting Blood Glucose | Candidate Predictor | 1,292 | 3.12% | Imputed |
| Body Temperature | Candidate Predictor | 1,103 | 2.66% | Imputed |
| Diastolic Pressure | Candidate Predictor | 873 | 2.11% | Imputed |
| Systolic Pressure | Candidate Predictor | 892 | 2.15% | Imputed |
| BMI | Candidate Predictor | 296 | 0.71% | Imputed |
| Urine Microalbumin | Candidate Predictor | 2,088 | 5.04% | Imputed |
| Complication Indicators | State Identification | - | - | Complete |
| Survival Time | Outcome | - | - | Excluded If Missing |
| Event Indicator | Outcome | - | - | Excluded If Missing |
| State | MDP State | - | - | Excluded If Missing |
| Action | MDP Transition Action | - | - | Excluded If Missing |
| Note: Missing proportions are reported at the patient level, with the 41,398-patient Nanjing analytic cohort as the denominator; the risk-prediction models were subsequently fitted to person-year records. The variables shown are selected core variables for which missingness was summarized and do not constitute the complete candidate-predictor set. After prespecified exclusions and one-hot encoding, 124 candidate design predictors entered the LASSO-Cox modeling procedure. Missingness was calculated before imputation. | | | | |

Appendix Figure 1. Workflow for Missing Data Assessment and Random-Forest-Based Imputation

**Appendix 5**

**ICD-10 Healthcare Security Version Coding Algorithm for Diabetes-Related Complication States**

Appendix Table 19. Operational Definitions of MDP Health States Based on the ICD-10 Healthcare Security Version

| MDP State | Health State | Operational Definition | ICD-10 Healthcare Security Version Code Category | State Assignment Rule |
| --- | --- | --- | --- | --- |
| S0 | No recorded diabetes-related complication | Type 2 diabetes with no recorded ICD-10-coded diabetic nephropathy, retinopathy, peripheral neuropathy, lower-extremity arterial disease, or diabetic foot before or during the monthly cycle. | No qualifying code from S1-S4 categories. | Assigned when no S1-S4 qualifying complication code was recorded. |
| S1 | Diabetic nephropathy | Presence of a structured ICD-10-coded diagnosis indicating type 2 diabetes with renal complications or diabetic nephropathy. | E11.2* and corresponding ICD-10 Healthcare Security Version extension codes for type 2 diabetes with renal complications or diabetic nephropathy. | Assigned when the first qualifying renal-complication code was recorded. |
| S2 | Diabetic retinopathy | Presence of a structured ICD-10-coded diagnosis indicating type 2 diabetes with ophthalmic complications or diabetic retinopathy. | E11.3* and corresponding ICD-10 Healthcare Security Version extension codes for type 2 diabetes with ophthalmic complications or diabetic retinopathy. | Assigned when the first qualifying ophthalmic-complication code was recorded. |
| S3 | Diabetic peripheral neuropathy | Presence of a structured ICD-10-coded diagnosis indicating type 2 diabetes with neurological complications or diabetic peripheral neuropathy. | E11.4* and corresponding ICD-10 Healthcare Security Version extension codes for type 2 diabetes with neurological complications or diabetic peripheral neuropathy. | Assigned when the first qualifying neurological-complication code was recorded. |
| S4 | Lower-extremity arterial disease and diabetic foot | Presence of a structured ICD-10-coded diagnosis indicating type 2 diabetes with peripheral circulatory complications, lower-extremity arterial disease, diabetic foot, foot ulcer, or diabetes-related gangrene. | E11.5* and corresponding ICD-10 Healthcare Security Version extension codes for type 2 diabetes with peripheral circulatory complications or diabetic foot; additional mapped diabetic-foot codes when applicable. | Assigned when the first qualifying peripheral-circulatory or diabetic-foot code was recorded. |
| S5 | Death | Death recorded in the public health management system or linked mortality record. | Mortality record rather than ICD-10 disease code. | Assigned from the month of death and modeled as an absorbing state. |
| Note: ICD-10 Healthcare Security Version refers to the ICD-10 coding dictionary used by the National Healthcare Security Administration of China. The asterisk indicates that all subcodes and extension codes under the corresponding ICD-10 Healthcare Security Version category were included if their diagnosis names matched the specified complication category. LEAD, lower-extremity arterial disease; MDP, Markov decision process. The state indices in this appendix were used consistently for transition probabilities, health utility values, cost parameters, sensitivity analyses, and all MDP computations. | | | | |

Appendix Table 20. State-to-Parameter Mapping Used in the MDP Implementation

| MDP State | Code Label | Health State | Utility Parameter | Utility Value | Cost Parameter | Annual Complication-Related Cost, CNY/year |
| --- | --- | --- | --- | --- | --- | --- |
| S0 | S0_no_complication | No recorded diabetes-related complication | $\text{u}_{\text{0}}$ | 0.868 | $c_{comp,0}$ | 0 |
| S1 | S1_kidney | Diabetic nephropathy | $\text{u}_{\text{1}}$ | 0.753 | $c_{comp,1}$ | 12,000 |
| S2 | S2_retinopathy | Diabetic retinopathy | $\text{u}_{\text{2}}$ | 0.830 | $c_{comp,2}$ | 4,500 |
| S3 | S3_neuropathy | Diabetic peripheral neuropathy | $\text{u}_{\text{3}}$ | 0.769 | $c_{comp,3}$ | 5,500 |
| S4 | S4_lower_limb_foot | Lower-extremity arterial disease and diabetic foot | $\text{u}_{\text{4}}$ | 0.692 | $c_{comp,4}$ | 8,000 |
| S5 | S5_death | Death | $\text{u}_{\text{5}}$ | 0.000 | $c_{comp,5}$ | 0 |
| Note: This mapping corresponds to the state-label, utility, and state-specific complication-cost arrays used in the MDP implementation. c_comp,s_ denotes the annual complication-related cost for health state s. For S0 and S5, c_comp,0_=0 and c_comp,5_=0. The annual baseline diabetes management cost c_base,i_ was modeled separately and was not part of the state-specific complication-related cost array shown in this table. The same state-to-parameter mapping was used in transition-probability estimation, MDP policy optimization, cost-effectiveness analysis, deterministic sensitivity analyses, and ICD-10 state assignment. | | | | | | |

Appendix Table 21. Rules for Identifying Prevalent and Incident Diabetes-Related Complications

| **Item** | **Definition Used in This Study** | **Operational Rule** |
| --- | --- | --- |
| Baseline prevalent complication | A complication already present at model entry. | Any qualifying ICD-10 Healthcare Security Version code for S1-S4 recorded before or at the first monthly cycle. |
| Incident complication | A newly observed complication during follow-up. | The first qualifying ICD-10 Healthcare Security Version code for S1-S4 recorded after model entry among patients who had not previously been assigned to that complication category. |
| No recorded complication | No qualifying complication code was recorded. | Patients remained in S0 if no qualifying ICD-10 code for S1-S4 was recorded before or during the corresponding monthly cycle. |
| Complication persistence | Complication status was treated as nonreversible. | Once a patient entered S1-S4, reversal to S0 was not allowed in the model. |
| Death | Death during follow-up. | Death was identified from mortality records and modeled as the absorbing state S5. |
| Patients with no analyzable longitudinal records | Patients without valid follow-up information. | Excluded from the analytic cohort if longitudinal follow-up was unavailable because of relocation, transfer out of the local public health management system, or incomplete or invalid records. |
| Note: These rules apply to MDP state assignment. The incident-complication risk model used a separate person-year risk set that excluded patients with prevalent complications in the first eligible year and all post-event person-years. | | |

Appendix Table 22. State-Assignment Algorithm for Patients With Multiple Qualifying Codes

| Scenario | State-Assignment Rule |
| --- | --- |
| Only one qualifying complication code was recorded before or during the monthly cycle. | Assigned to the corresponding complication state S1-S4. |
| More than one qualifying complication category was recorded on different dates. | Assigned according to the earliest recorded qualifying complication category. |
| More than one qualifying complication category was recorded within the same monthly cycle. | Assigned according to the earliest diagnosis date within that monthly cycle. |
| More than one qualifying complication category had the same diagnosis date. | Assigned according to the first structured diagnosis record in the EHR data pipeline. |
| Death occurred in the same cycle as a complication code. | Assigned to S5 from the month of death because death was modeled as the absorbing state. |
| A later record showed a different complication category after assignment to S1-S4. | The patient remained in the originally assigned complication state unless death occurred, because complication reversal or switching between complication states was not allowed in the base-case MDP model. |

Appendix Table 23. ICD-10 Healthcare Security Version Code Ranges Used to Identify Diabetes-Related Complications

| Complication Category | Primary ICD-10 Healthcare Security Version Code Prefix | Diagnosis Names Included | Diagnosis Names Excluded |
| --- | --- | --- | --- |
| Diabetic nephropathy | E11.2* | Type 2 diabetes with renal complications; diabetic nephropathy; diabetic kidney disease; diabetes-related renal complication. | Non-diabetic chronic kidney disease without a diabetes-related ICD-10 diagnosis code. |
| Diabetic retinopathy | E11.3* | Type 2 diabetes with ophthalmic complications; diabetic retinopathy; diabetes-related retinal disease. | Non-diabetic retinopathy or other ophthalmic disease without a diabetes-related ICD-10 diagnosis code. |
| Diabetic peripheral neuropathy | E11.4* | Type 2 diabetes with neurological complications; diabetic peripheral neuropathy; diabetes-related neuropathy. | Non-diabetic neuropathy without a diabetes-related ICD-10 diagnosis code. |
| Lower-extremity arterial disease and diabetic foot | E11.5* | Type 2 diabetes with peripheral circulatory complications; diabetic lower-extremity arterial disease; diabetic foot; diabetic foot ulcer; diabetes-related gangrene. | Peripheral arterial disease, foot ulcer, or gangrene not coded as diabetes-related in the ICD-10 Healthcare Security Version dictionary. |
| Multiple or unspecified diabetic complications | E11.7* and E11.8* | Used only when the structured diagnosis name or local extension code could be mapped to one of the prespecified complication categories S1-S4. | Not assigned to S1-S4 if the record did not specify renal, ophthalmic, neurological, peripheral circulatory, or diabetic-foot involvement. |
| Type 2 diabetes without complications | E11.9* | Type 2 diabetes without recorded complication. | Not considered a complication state. |
| Note: Code prefixes were applied to all corresponding subcodes and extension codes in the ICD-10 Healthcare Security Version dictionary. E11.7* and E11.8* were not directly assigned to a specific MDP complication state unless the structured diagnosis name or local extension code explicitly indicated renal, ophthalmic, neurological, peripheral circulatory, or diabetic-foot involvement. Records with nonspecific complication codes that could not be mapped to S1-S4 were not used to define incident complication states in the base-case model. | | | |

Appendix Table 24. Secondary Analyses of the Risk Prediction Models: lambda.min Tuning Rule and Cross-Sectional Discrimination

| Outcome | Analysis | Predictors retained | Apparent C-index | Conditional patient-level bootstrap-corrected C-index | External C-index |
| --- | --- | --- | --- | --- | --- |
| Incident complication | Primary (lambda.1se) | 1 | 0.777 | 0.777 | 0.778 |
| Incident complication | Secondary (lambda.min) | 65 | — | — | 0.752 |
| Mortality | Primary (lambda.1se) | 7 | 0.768 | 0.767 | 0.812 |
| Mortality | Secondary (lambda.min) | 95 | — | — | 0.833 |
| Note: The lambda.1se rule was prespecified before model fitting as the primary tuning rule; the lambda.min rule is reported as a secondary analysis. Models were not selected on the basis of external validation performance. Apparent and conditional patient-level bootstrap-corrected C-indices are reported only for the primary analyses; comparison across tuning rules is based on the external C-index, which is the quantity of interest for these secondary analyses. Cross-validation folds were assigned at the patient level, and the conditional optimism bootstrap resampled patients and refitted the Cox model conditional on the LASSO-selected predictor set. Four categorical variables selected in the lambda.min models (occupation, marital status, medical payment type, and blood group) were structurally unavailable in the Shanghai dataset. For the secondary lambda.min external-discrimination analyses only, these variables were fixed at their development-cohort reference levels; no additional missing-value imputation was performed during the lambda.min model-fitting or external-scoring stage because predictor imputation had been completed separately during cohort-specific preprocessing. Neither primary lambda.1se model included these variables, so the primary external validation results were unaffected. In cross-sectional analyses restricted to a single follow-up year, in which each patient contributed one observation, duration of diabetes alone gave a C-index of 0.790 in the second follow-up year (32,923 patients, 1,194 events) and 0.768 in the third follow-up year (25,564 patients, 1,383 events). | | | | | |
